# Consumer Expectations for Cream Cheese: A Category Appraisal Study in the United Kingdom with Dairy and Plant-Based Variants in Various Flavours

**DOI:** 10.3390/foods14030445

**Published:** 2025-01-29

**Authors:** Sara R. Jaeger, Sok L. Chheang, Joachim J. Schouteten

**Affiliations:** 1 Department of Food Science, Aarhus University, 8200 Aarhus, Denmark; 2 The New Zealand Institute for Plant and Food Research Limited, 120 Mount Albert Road, Auckland 1025, New Zealand; sokleang.chheang@plantandfood.co.nz; 3 Department of Agricultural Economics, Ghent University, Coupure Links 653, 9000 Ghent, Belgium; joachim.schouteten@ugent.be

**Keywords:** consumer research, plant-based foods, cheese, online survey

## Abstract

The purpose of the present research was to deliver new knowledge of consumer expectations for plant-based (PB) alternatives to cream cheese (PBCCA) by conducting a category appraisal study. Around 1100 consumers from the United Kingdom (UK) who followed omnivore and flexitarian diets participated in an online survey. They evaluated 13 written stimuli presented as product names encompassing dairy cream cheese and PBCCAs in different flavours (original/natural, garlic/herb, salmon, strawberry, chocolate) and different product formulations (low-fat, lactose-free). A multi-response approach was used that obtained sensory, emotional, conceptual, and versatility product evaluations. The research findings, which matched predictions, showed: (1) negative product expectations for PBCCAs replicated across flavour variants, providing evidence of a systematic PB effect relative to cream cheese; (2) sensory and non-sensory drivers of expected product liking resembled those established for the cream cheese category with actual product experience; (3) sensory and non-sensory drivers of expected product versatility strongly resembled those of expected product liking; (4) groups of consumers existed with different preferences, including flavour and product type preferences (dairy, PBCCA); and (5) negative product expectations for PBCCAs translated to a stated behavioural preference for cream cheese over PBCCA, and dairy-based samples were chosen over their PB counterparts regardless of flavour. The category appraisal approach confirmed the systematic negative appeal of PBCCAs relative to their dairy counterparts. This was in line with findings from past research on other PB alternative foods and invites a rethinking of the appeal of this class of products to participants in this research, who represent a large segment of UK consumers.

## 1. Introduction

### 1.1. Consumer Expectations for Plant-Based Alternative Foods

The demand for plant-based (PB) foods has risen in recent years, driven by growing concerns over environmental sustainability, personal health, and animal welfare [1,2,3]. The global food system is responsible for significant greenhouse gas emissions, resource depletion, and biodiversity loss, contributing about 30% of global emissions, with animal agriculture accounting for a substantial share [4,5,6]. Consumers are starting to seek alternatives that reduce environmental impact, and PB alternative foods offer a more sustainable option, generally requiring less water and land to produce than animal-based products [7,8]. Additionally, there is evidence linking PB diets to improved health outcomes, including lower risks of chronic diseases such as heart disease, obesity, and type 2 diabetes [9,10,11]. This convergence of ethical, health, and environmental motivations has spurred innovation within the food industry, leading to a growing variety of PB foods that mimic traditional animal-derived products [12,13]. The demand for these PB alternatives highlights a shift in dietary preferences and underscores the importance of understanding consumer expectations as the industry works to improve further and expand its product ranges of PB alternative foods.

Past studies demonstrate that expectations significantly influence food acceptance [14,15]. Consumer expectations for PB alternative foods are shaped by sensory attributes like taste, texture, and aroma and by non-sensory drivers, including emotions, product associations, and values [16,17]. Sensory qualities remain paramount, as taste and texture directly influence enjoyment and acceptance of these products, with low expected sensory quality of PB alternative foods being a main consumption barrier [18]. However, non-sensory drivers also significantly affect perceptions and purchase intentions. Emotional responses, such as feeling good about making sustainable choices or supporting animal welfare, can provide additional insights into the appeal of PB alternative foods [16,19]. Additionally, consumers have unique conceptual expectations of PB foods, such as not being ‘natural’ or minimally processed, which may influence acceptance and trust [20]. Meeting consumer expectations for PB alternative foods involves significant challenges, particularly in balancing the aforementioned sensory and non-sensory expectations. Understanding these sensory and non-sensory expectations is essential for aligning product attributes with consumer desires and supporting the adoption of PB food alternatives.

### 1.2. Research Aim and Expected Findings

The aim of the present research was to expand existing knowledge of consumer expectations for PB alternative foods. Focus was directed to the cheese category, which is relevant because of its importance in diet and because PB alternatives to cheese are less studied than other PB alternative product categories, notably meat [21,22,23,24,25] and milk [26,27]. The scientific novelty of the work was further strengthened by performing a category appraisal study, which compared diverse samples within a single product category. This strategy extended past approaches of comparing product variants made from different PB ingredients with one another [28] or comparing animal-based and PB variants with each other [16,29,30,31] and mandated a sample selection strategy that included flavour variants and samples with other intrinsic characteristics representative of the focal category (e.g., low salt, low-fat, lactose-free). The cream cheese category provided the needed diversity (see Section 2.2 for more detail).

Several predictions about the study’s expected findings (EFs) were made and are elaborated below.

1. Negative product expectations for PBCCAs will replicate across flavour variants, providing evidence of a systematic PB effect relative to cream cheese (EF1).
2. Sensory and non-sensory drivers of expected product liking in the cream cheese category will resemble those based on actual product experience (i.e., product tasting) (EF2).
3. Sensory and non-sensory drivers of expected product versatility can be established, and they will strongly resemble those of expected product liking (EF3).
4. Groups of consumers exist with different preferences, including flavour and product type preferences (dairy, PBCCA) (EF4).
5. Negative product expectations for PBCCAs will translate to a behavioural preference for cream cheese over PBCCA, and dairy-based samples will be chosen over their PB counterparts regardless of flavour. A higher choice probability may be observed if consumer segments with more positive PBCCA product expectations exist (EF5).

#### 1.2.1. Expected Finding 1

Past research has indicated that consumers often have lower expectations for PB food alternatives than traditional animal-based counterparts, associating them with inferior taste, texture, or sensory satisfaction [16,32,33]. While research has shown that consumers often approach PB dairy alternatives with lower expectations [32,34], little is known about whether these biases affect perceptions of different flavour options within the same product category. The lower expectations are further reinforced by familiarity biases, as consumers accustomed to traditional dairy-based cream cheese often anticipate that plant-based alternatives will not meet the same standards in key sensory attributes [30]. As a result, expectations of PBCCAs are likely to remain systematically lower, regardless of flavour variety (EF1), suggesting that consumer biases may be deeply rooted and challenge the acceptance of PB variants more broadly.

#### 1.2.2. Expected Finding 2

Recent research has increasingly focused on expanding beyond traditional sensory evaluation to gain a more comprehensive understanding of consumer food choices [35,36,37]. This shift has led to a substantial body of work examining non-sensory drivers, such as emotional responses and consumer conceptualisations of food products. However, studies specifically addressing non-sensory drivers in the context of PB food alternatives remain limited [30]. Given that consumer expectations are often anchored in prior experiences with similar products, including usage frequency and familiarity with PB foods [34], it is anticipated that expected sensory and non-sensory drivers will align with actual product experience (EF2).

#### 1.2.3. Expected Finding 3

Cream cheese is a versatile product used in various culinary applications, from spreading on bread to enhancing sauces, dips, and desserts. Its ability to fit diverse contexts stems from sensory qualities like a smooth, spreadable texture and mild flavour, which make it adaptable across savoury and sweet dishes [30]. Prior research by Jaeger et al. [30] evaluating two PBCCAs and two dairy cream cheeses during a taste test found that the dairy cream cheeses were seen as more versatile than PBCCAs by the consumers. Moreover, sensory and non-sensory drivers were established and linked to the products, suggesting a close relationship between liking and versatility in consumer perceptions [30]. Previous research has demonstrated a strong correlation between the perceived versatility of food products and consumer liking [38,39] (EF3), but sensory drivers of versatility have not previously been established.

#### 1.2.4. Expected Finding 4

In examining consumer expectations for cream cheese and its PB alternatives, it is anticipated that distinct consumer segments will emerge, characterised by varied flavour and product type preferences, including preferences for dairy versus PB options. Previous research has identified two main consumer segments: one group showing equal preference for PBCCAs and dairy cream cheeses, and another favouring dairy cream cheese [30]. Additional studies on PB dairy alternatives further support the likelihood of distinct consumer segments for PB and dairy products, with segmentation patterns potentially differing across product categories (e.g., milk, meat, fish, cheese) [20,40]. While segmentation based on flavour preferences has not yet been investigated specifically for PBCCA, this differentiation is plausible (EF4), as research frequently reports individual differences in flavour preferences across food products and beverages [41].

#### 1.2.5. Expected Finding 5

Research suggests that PB dairy substitutes are often met with lower consumer expectations, particularly regarding sensory attributes such as taste, texture, and perceived authenticity, which may predispose consumers to favour traditional dairy options when given a choice [32,34]. These inherent biases can lead consumers to prefer dairy-based products over PB alternatives even when flavour profiles are comparable, reinforcing a general preference for conventional dairy options. However, evidence also points to consumer segments with more favourable attitudes toward PB products such as PBCCAs [20,30]. These consumers tend to have more positive product expectations, which may increase the probability of choosing PBCCAs, supporting the EF5 notion that product expectations significantly shape choice behaviour across distinct consumer groups.

## 2. Materials and Methods

### 2.1. Participants

Adults living in the United Kingdom (UK) participated. They had self-registered on a database managed by a web panel provider with ISO 20252:2019 accreditation [42] and indicated an interest in and willingness to complete surveys. Only people with self-declared proficiency in English could participate.

Eligible participants were product category users, excluding vegans (who would not consume dairy cream cheese) and people with major allergies. Thus, participants were excluded if they had not eaten cream cheese in the last 12 months, answered in the affirmative to the statement ‘I don’t eat meat, and I don’t use products of animal origin’, or self-reported major food allergies/intolerances (incl. gluten, nuts, soy, eggs, dairy, corn, wheat, and seafood).

Interlocking quotas for men (50%) and women (50%) across two age groups (18–45 years old (50%) and 46–69 years old (50%) were imposed. The overall sample was broadly nationally representative and diverse across various characteristics such as household income, household members, educational attainment, and place of living (Table 1). Refer to Part SM1 in the Supplementary Materials for additional sample information and a comparison against the most recent UK Census data for selected participant characteristics.

### 2.2. Product Category and Research Stimuli

#### 2.2.1. Product Category

Cheese is a major product category globally and highly developed in most markets [43,44,45]. In large Western markets like the European Union, the United States, and Canada, per capita intake is around 14.6 to 20.5 kg per year [46]. Because the cheese category is extremely diverse, with hundreds, if not thousands, of different types of cheese [47], it was decided to focus on one segment within this diverse category. From several options, cream cheese was selected. While small compared with the overall cheese category, the cream cheese market is growing [48]. More importantly, in the context of the present research aim, cream cheese is a diverse category with many flavours and several texture variants [49].

Traditionally, cream cheese was a plain product with no additional flavourings, made simply by adding lactic acid bacteria to pasteurised milk and cream, which thickens and acidifies the mixture. The milk curds are gently heated and strained to remove excess whey. Finally, the cheese is blended until creamy and spreadable [50]. Responding to consumer demands, cream cheese formulations with a lower fat content [51] and without lactose also exist. Plant-based alternatives to cream cheese (PBCCA) are also becoming more widely available.

#### 2.2.2. Research Stimuli (Product Names)

Table 2 lists the 13 product stimuli included in the study. These were selected to span the focal category and be suitable for a category appraisal study. For flavour variants, market leaders were represented by the flavours original (hereafter original/plain) and garlic and herb (hereafter garlic/herb) [49]. Niche products were low-fat and lactose-free variants of these two flavours. Further niche products were represented by three flavours: strawberry, milk chocolate, and salmon. Reflecting that different PB ingredients can be used in cream cheese product formulation [49,52], almond and oats and coconut oil variants (original/plain flavour) were included in the sample set.

The subset of six samples (marked with asterisks in Table 2) followed a 3 × 2 factorial (F1: cream cheese, low-fat cream cheese, PBCCA; F2: original/plain flavour, garlic/herb flavour). For this 6-sample subset, more extensive product evaluations were obtained (EF2, EF3).

### 2.3. Survey Measures and Data Collection

#### 2.3.1. Product Responses

The responses collected for all product stimuli were the stated expectations of product liking, emotional product associations, and product versatility.

Expected product liking was obtained through Case 1 (object-based) best–worst scaling (BWS) [53]. In this task, participants were presented with thirteen sets of four stimuli per a balanced incomplete block design (BIBD plan 11.22) [54]. Participants were instructed to imagine eating different types of cream cheese and non-dairy/plant-based alternatives and to indicate their expected liking/disliking based on the product description. For each set of four names (thirteen sets for the full task), the one product they expected to like most and the one they expected to like least had to be selected (see Part SM2 in the Supplementary Materials for a screenshot of the task).

Using a CATA (check-all-that-apply) question format Ares and Jaeger [55], the expected emotional product associations were obtained using 12 pairs of emotion words (Table 3) from the valence × arousal circumplex-inspired emotion questionnaire [56]. Expected versatility was rated on a 7-point scale with end-point anchors (1 = ‘low versatility of use/few different uses’; 7 = ‘high versatility of use/many different uses’). Participants were instructed to consider expected versatility, i.e., whether they expected the product to have few vs. many uses.

Next, the six products marked with * in Table 2 were evaluated for sensory product expectations and expected conceptual product associations (Table 3). The CATA question for sensory terms comprised 20 descriptors based on the findings of Jaeger et al. [30] and Foguel et al. [51]. For conceptual descriptors, the CATA question comprised 18 descriptors based on [57]. Contrary to emotional product associations, which were specifically aligned with valence and arousal, conceptual terms are broader and capture diverse product associations that extend beyond emotional domains [36]. In this part of the study, each participant evaluated only a single product name to reduce the response burden (Part SM3 in the Supplementary Materials details the number of participants that evaluated each stimulus). In line with past research by Jaeger and colleagues in the dairy category [17,20,30,31,40,55,58], definitions of CATA terms were not provided.

Behavioural intent for product choice was gauged in the following question: Please imagine that you were given the option to take home one of the cream cheeses or cream cheese alternatives described in the survey as a thank you for your participation. Participants had to choose between the options ‘Dairy version (any flavour of your choice)’ and ‘Plant-based alternative (any flavour of your choice)’.

Stated liking for, respectively, ‘cream cheese (dairy)’ and ‘plant-based alternative to cream cheese (non-dairy)’ was rated on fully labelled 9-point hedonic scales from 1 = ‘dislike extremely’ to 9 = ‘like extremely’. The frequency of consumption was collected on a fully labelled 9-point scale with the following categories: 1 = ‘never or less than once a year’; 2 = ‘1–3 times a year’; 3 = ‘every 2–3 months’; 4 = ‘once a month’; 5 = ‘2–3 times a month’; 6 = ‘once a week’; 7 = ‘2–4 times a week’; 8 = ‘5–6 times a week’; and 9 = ‘once daily or more often’.

#### 2.3.2. Participant Characteristics

Toward the end of the survey, participants completed the 9-item dietary habits question from De Backer and Hudders [59] and standard socio-economic profiling questions (e.g., household size, household income, education attainment, employment status).

#### 2.3.3. Data Collection

From a location of their choosing, participants could complete the survey using any internet-connected electronic device. Data collection took place in April and May 2024 following careful revision of the test links and the evaluation of the responses from ~10% of the sample to ensure that the survey performed as expected.

Randomisation was used during data collection to mitigate order effects. Within the BWS tasks, the presentation order of the choice sets and statements within each set were randomised across participants. For the other response types, the 13 product stimuli were presented monadically and in randomised order across participants. For the CATA question, the presentation order of terms was randomised both across stimuli and participants, according to Ares and Jaeger [55].

The data used for this research were collected as part of a more extensive survey. Other tasks/responses are not considered further for lack of relevance.

### 2.4. Data Cleaning and Analysis

#### 2.4.1. Data Cleaning

When collecting data online, quality assessment and cleaning are paramount, owing to widespread reports of problems with low quality [60,61]. Part SM4 in the Supplementary Materials features a data quality statement per Jaeger and Cardello [60] for the 1222 responses in the data delivered by the commercial research provider.

Of these 1222 participants, another 129 were excluded before analyses based on two study-specific indices of low data quality in the product evaluation responses. One was a flatline criterion [62] imposed on the ratings of expected versatility, which 28 participants failed because they gave the same response to all 13 product stimuli. The second criterion was imposed on the BWS responses that captured participants’ expected product liking. Following the lead of Llobell et al. [63], who have shown that the BWS data collected online need to be quality validated, the Normalised Error Variance (EVN) index introduced by those authors was used since it is specific to Case 1 BWS data. The index is based on Louviere et al. [53] and measures the variance in each participant’s BWS choices; it can range between 0 and 1, with higher values indicating less variance and more consistency. If a participant selects the same ‘best’ and ‘worst’ options every time they are available, EVN = 1. The index is easily calculated based on B-W (best-minus-worst) scores using the equation in Part SM5 of the Supplementary Materials. Based on the findings of Llobell et al. [63], respondents with EVN < 0.5 were excluded (Part SM6 in the Supplementary Materials provides a numerical example for further explanation). Imposing this criterion further reduced the sample size to *n* = 1093. For the 6-sample subset, between 179 and 186 participants evaluated each of the stimuli.

#### 2.4.2. Converting BWS Responses to B-W Scores

For expected product liking, the BWS choice responses were converted into B-W scores (also called BWS scores) for each task. They are derived from a simple count analysis, which for each object (i.e., product name or food choice motive) are the ‘best’ frequency counts minus the ‘worst’ frequency counts.

The B-W scores for expected liking could range between +4 and −4, with +4 indicating that a product was always chosen as ‘best’ (i.e., expected to be liked most) when presented with other products. Conversely, −4 indicated that a product was always chosen as ‘worst’ (i.e., expected to be liked least) when presented with other products. B-W = 0 indicated that a product was selected as ‘best’ the same number of times that it was selected as ‘worst’, meaning that the expected liking was neither above nor below average. B-W scores have distance measurement properties, making them suitable for parametric data analysis [64].

#### 2.4.3. Data Analysis

For EF1, aggregate-level data analyses were performed with the 1093 retained participants. Generalised linear modelling was applied to the B-W scores for expected liking, with Tukey’s HSD for post hoc tests. The same was performed for expected versatility. For CATA data, frequency tables were derived and subjected to Correspondence Analysis using chi-square distances.

Penalty/lift analysis [65] (EF2, EF3) was used to determine the mean impact of term citation on expected liking and expected versatility. These analyses were performed only on the 6-sample subset, progressing one CATA term at a time (20 sensory terms, 12 emotional terms, 18 conceptual terms). Using 2-sample *t*-tests, the differences in the mean liking/versatility when a focal term was selected vs. unselected were tested to see if they differed significantly from zero. Caution about a test result was observed when fewer than 100 participants (of 1093) had used a term (~9.1%).

For EF4, consumer segments based on expected product liking were derived using hierarchical cluster analysis (Ward’s method, Euclidian distances), which is widely used in sensory and consumer science. A consolidation (or trimming) protocol was applied to the retained clusters to ensure that people within a cluster were sufficiently similar to each other and the cluster average. Based on the findings of Hasted [66], participants were excluded if the correlation between their mean liking scores and the cluster mean liking scores was less than 0.4. This reduced the sample size from 1093 to 947 for the results pertaining to consumer segmentation. Three clusters were retained, based on the dendrogram (Part SM7 in the Supplementary Materials).

For EF5, the hypothetical take home choice task, logistic regression was used to estimate the effects of stated liking, frequency of consumption, and dietary preferences on the likelihood of selecting a PPBCA product.

All analyses were performed in XLSTAT [67], which is a commercially available programme that is implemented as an add-on to Excel. A 5% significance level was used for inference testing.

## 3. Results

### 3.1. Product Expectations Across Total Consumer Sample (EF 1)

#### 3.1.1. Expected Product Liking

Of the thirteen product stimuli included in this research (Table 2), the eight product variants that were expected to be liked most, on average, were four pairs of cream cheeses with garlic/herb flavour and original/plain flavour, with the latter flavour always being expected to be liked less. The regular product pair had the highest B-W scores, followed by the low-fat pair, the lactose-free pair, and the PB pair (made from almonds and oats). The five products that consumers, on average, expected to like the least were cream cheeses with niche flavours (salmon, milk chocolate, strawberry) and two PBCCA (original/plain flavour made from coconut oil, and strawberry flavour).

#### 3.1.2. Expected Product Emotional Associations

**Figure 1:**
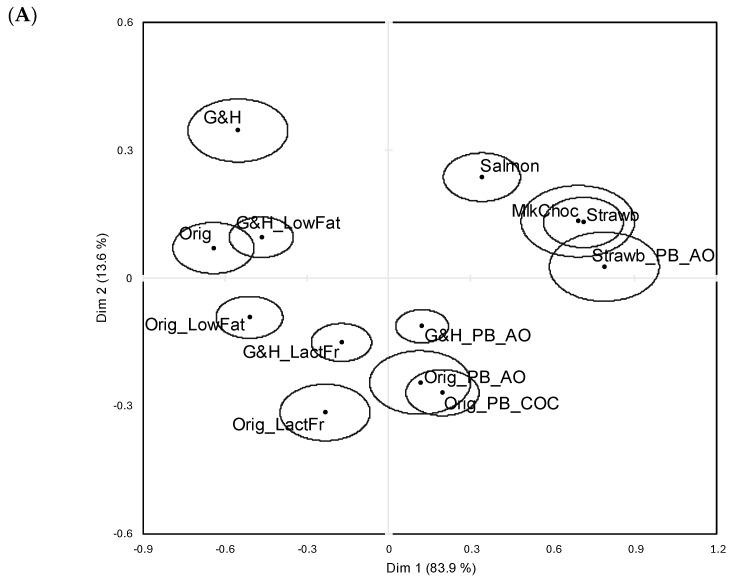

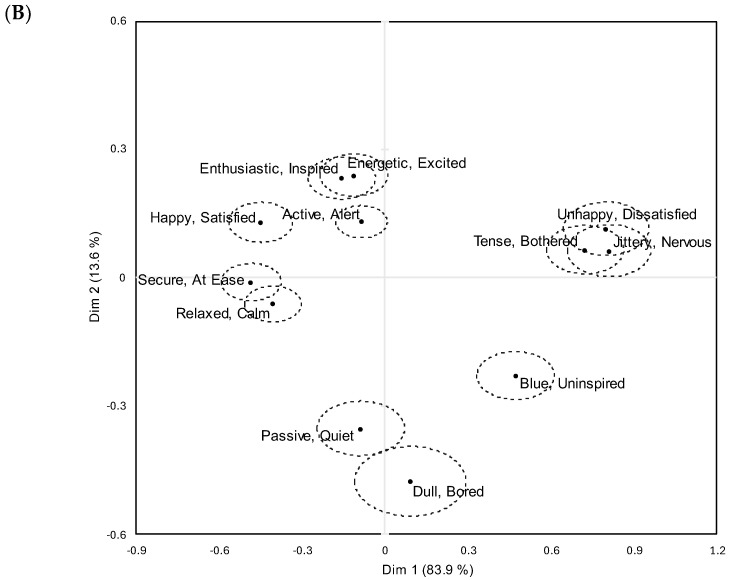
Results for EF1. Two-dimensional solutions (Dim 1 (83.9%) and Dim 2 (13.6%)) following Correspondence Analysis based on expected emotional associations to 13 cream cheese product stimuli included in research. (**A**) Product space with 95% confidence ellipses around average sample positions. (**B**) Variable space with 95% confidence ellipses around average variable positions. Sample abbreviations: Orig = cream cheese, original/plain flavour; Orig_LowFat = cream cheese, original/plain flavour, low-fat; Orig_PB_AO = cream cheese, original/plain flavour, plant-based alternative (almonds and oats); G&H = cream cheese, garlic and herbs flavour; G&H_LowFat: cream cheese, garlic and herbs flavour, low-fat; G&H_PB_AO: cream cheese, garlic and herbs flavour, plant-based alternative (almonds and oats); Orig_PB_COC: cream cheese, original plain flavour, plant-based alternative (coconut oil); Orig_LactFr: cream cheese, original/plain flavour, lactose-free; G&H_LactFr: cream cheese, garlic and herbs flavour, lactose-free; Strawb: cream cheese, strawberry flavour; Strawb_PB_AO: cream cheese, strawberry flavour, plant-based alternative (almonds and oats); MlkChoc: cream cheese, milk chocolate flavour; Salmon: Cream cheese, salmon flavour.

A two-dimensional solution, which explained nearly 100% of the inertia in the data, was retained. The solution was dominated by the first dimension, which was valence-driven. Of the thirteen product stimuli, the six with the highest expected liking scores were positioned furthest to the left of Figure 1A, while the three product stimuli expected to be least liked were positioned furthest to the right of Figure 1A. This corresponded with the emotional associations (Figure 1B), which for the most liked samples were ‘happy/satisfied’, ‘enthusiastic/inspired’, and/secure/at ease’, and for the least liked samples were ‘unhappy/dissatisfied’, ‘blue/uninspired’, and ‘tense/bothered’. The second dimension was related to emotional arousal, as seen by the axis being spanned by the word pairs ‘active/alert’ to ‘passive/quiet’ and ‘energetic/excited’ to ‘dull/bored’ (Figure 1B). This extended the insight for the six samples with the highest expected liking scores, since the variants with original/plain flavours were always positioned closer to the deactivated pole (i.e., the bottom half of Figure 1A). This pattern also held for the two flavour variants in the almond/oat PBCCA product pair. The emotional associations for the PBCCA in the original/plain flavour largely did not depend on the PB ingredient being almond/oat or coconut oil, as seen from the overlapping 95% confidence ellipses for these product stimuli names.

The Correspondence Analysis results revealed negative PBCCA expectations when the product flavours were garlic/herb and original/plain, as did the Cochran’s Q tests. Significant differences in product citation frequencies were observed for all emotion word pairs when the flavour was garlic/herb (i.e., dairy vs. PBCCA). For the original/plain flavour, significant product differences were observed for 11 of 12 emotion word pairs when the PBCCA variant was made with coconut oil and for 9 of 12 emotion word pairs when the PBCCA variant was made with almonds and oats. The exception was the strawberry-flavoured cream cheese. The 95% confidence ellipses for the cream cheese and PBCCA variants overlapped somewhat in Figure 1A, and in Cochran’s Q test, there were no significant differences for any of the 12 emotion word pairs.

#### 3.1.3. Expected Product Versatility

Table 2 also contained the EF1 results for expected product versatility. They mirrored those for expected liking and emotional associations, and a strong positive correlation existed between expected liking and expected versatility (r = 0.91, *p* < 0.0001). Of the thirteen product stimuli, the four with the highest expected versatility were those with the highest expected liking scores, and the same was observed for the three least liked/versatile products. One point of difference, however, seemed to be the tendency for the original/plain flavour variants to have higher versatility scores than the garlic/herb flavour variants.

#### 3.1.4. Product Expectations in Six-Sample Subset

**Figure 2:**
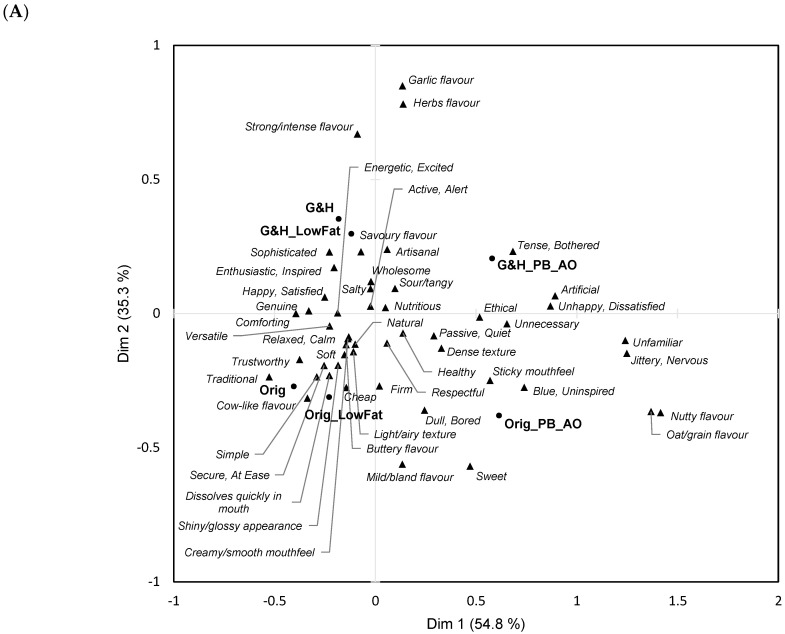

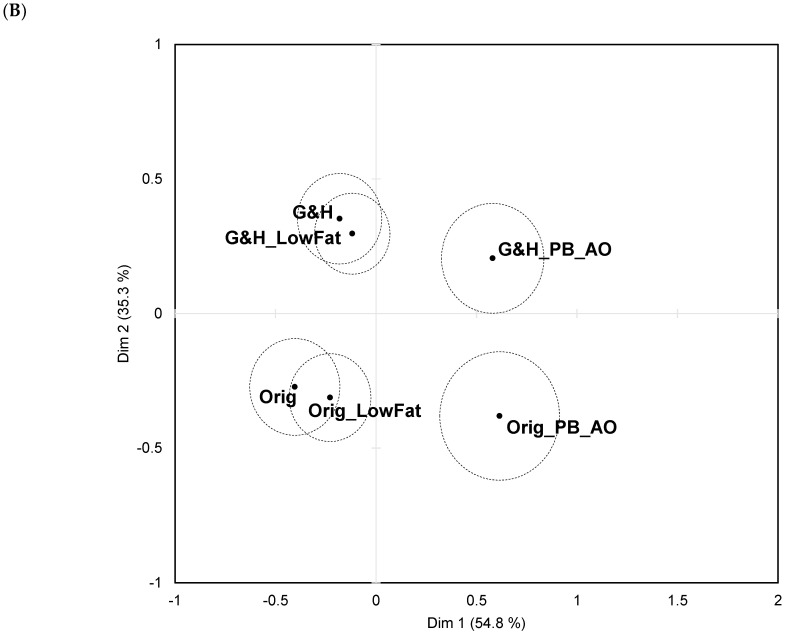
Results for EF1. Two-dimensional solutions (Dim 1 (54.8%) and Dim 2 (35.3%)) following Correspondence Analysis based on expected sensory, emotional, and conceptual associations to six stimuli in cream cheese product subset. (**A**) Biplot showing samples and terms (sensory, emotional, and conceptual). Supplementary Materials Part SM8 contains plot with all terms clearly labelled. (**B**) Variable space with 95% confidence ellipses around average variable positions. Sample abbreviations: Orig = cream cheese, original/plain flavour; Orig_LowFat = cream cheese, original/plain flavour, low-fat; Orig_PB_AO = cream cheese, original/plain flavour, plant-based alternative (almonds and oats); G&H = cream cheese, garlic and herbs flavour; G&H_LowFat: cream cheese, garlic and herbs flavour, low-fat; G&H_PB_AO: cream cheese, garlic and herbs flavour, plant-based alternative (almonds and oats).

Figure 2 shows the two-dimensional solution retained after Correspondence Analysis (CA) of the frequency table for the six-sample subset across the 50 sensory, emotional, and conceptual terms. It accounted for 90% of the inertia in the data and separated the two PBCCAs from the four cream cheeses along the first CA dimension. The garlic/herb cream cheeses were separated from those with original/plain flavours on the second CA dimension. The latter two samples were more strongly associated with ‘milk/bland flavour’ and deactivated emotions (‘passive/quiet’ and ‘dull/bored’) (Figure 2A). Unsurprisingly, the two garlic/herb samples were expected to have garlic and herb flavours and a more intense flavour. Other characteristics associated with the original/plain cream cheese samples were ‘cow-like flavour’, ‘traditional’, and ‘trustworthy’, whereas ‘unfamiliar’ and ‘jittery/nervous’ were frequently expected to be characteristic of the PBCCA samples. Other terms associated with PBCCAs and not with cream cheeses were ‘unnecessary’, ‘artificial’, and ‘unhappy/dissatisfied’. For completeness, a supplementary plot for Figure 2A with all terms clearly labelled is shown in Part SM8 of the Supplementary Materials. Additionally, two-dimensional solutions after CA of, respectively, sensory, emotional, and conceptual product expectations are provided in Parts SM9, SM10, and SM11 of the Supplementary Materials.

Overlapping 95% confidence ellipses around the average sample positions (Figure 2B) indicated that the two garlic/herb cream cheeses were similarly perceived, as were the two original/plain cream cheeses. The PBCCAs were perceived as different from one another, and from the two pairs of cream cheese samples.

### 3.2. Sensory, Emotional, and Conceptual Drivers of Expected Liking (EF2) and Expected Versatility (EF3) in Six-Sample Product Subset

For the subset of six samples with original/plain and garlic/herb flavours (Table 2), drivers of expected liking and versatility were obtained using penalty/lift analysis. The results are shown in Table 3 by the type of term (sensory, emotional, and conceptual).

#### 3.2.1. Drivers of Expected Liking

Expected sensory characteristics associated with a hedonic lift are the following, in order of decreasing mean impact from 1.2 to 0.5 scale points: ‘creamy/smooth mouthfeel’, ‘soft’, ‘savoury flavour’, ‘shiny/glossy appearance’, ‘strong/intense flavour’, ‘buttery flavour’, ‘garlic flavour’, ‘herbs flavour’, and ‘light/airy texture’. Conversely, expected sensory characteristics with a hedonic penalty were ‘nutty flavour’ (−1.4), ‘oat/grain flavour’ (−1.4), and ‘mild/bland flavour’ (−0.8). Penalties were also observed for ‘sticky mouthfeel’ and ‘sweet’, but the results must be considered somewhat cautiously because of low citation frequency for these expected sensory characteristics (indicated with ^#^ in the second column of Table 3).

When the product stimuli were associated with positive emotions (‘energetic/excited’, ‘enthusiastic/inspired’, ‘happy/satisfied’, ‘secure/at ease’, and ‘relaxed/calm’), there was a lift in expected liking (1.6 to 0.9 scale points) and, correspondingly, negative emotions (‘dull/bored’, ‘blue/uninspired’, ‘unhappy/dissatisfied’, ‘tense/bothered’, ‘jittery/nervous’) were associated with a penalty on expected liking (2.5 to 1.4 scale points). Owing to low citation frequency, only the result for ‘unhappy/dissatisfied’ (−2.5) was highly robust.

There were many significant effects on mean expected liking for conceptual product associations. The majority were positive (1.6 to 0.6 scale points): ‘comforting’, ‘traditional’, ’genuine’, ‘versatile’, ‘wholesome’, ‘trustworthy’, ‘simple’, ‘nutritious’, ‘natural’, and ‘sophisticated’. The result for the last of these terms may lack robustness because of low citation frequency, and the same caution applied to three conceptual terms that negatively affected mean liking (‘unnecessary’, ‘unfamiliar’, and ‘artificial’) (−2.0 to −1.5).

#### 3.2.2. Drivers of Expected Versatility

**Figure 3:**
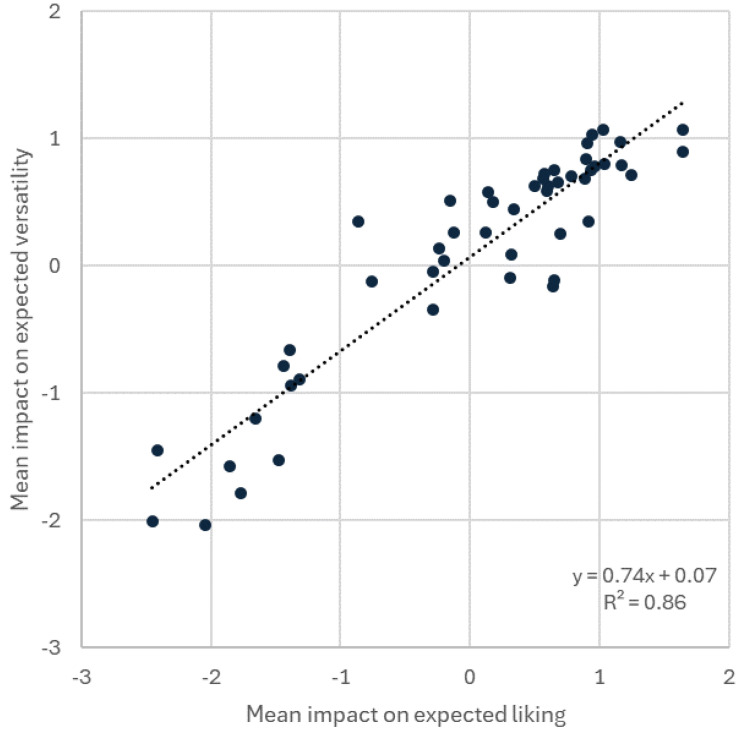
Results for EF3. Scatterplot of = mean impact on expected liking and expected versatility following penalty/lift analysis performed on six-sample cream cheese subset using all 50 sensory, emotional, and conceptual descriptors.

Table 3 gives the mean impacts on expected product versatility following penalty/lift analysis. This set of results was closely aligned with the penalty/lift results for expected liking, as seen in Figure 3, and the strong linear relationship (R^2^ = 0.86). Among the terms where citation frequency exceeded 100 people, as indicated by ^#^ in Table 3, notable deviations were three sensory terms with a significant mean impact on expected liking but not on expected versatility (‘garlic flavour’, ‘herbs flavour’, ‘mild/bland flavour’) and one conceptual term had a significant impact on mean expected versatility but not on expected liking (‘healthy’).

### 3.3. Consumer Segmentation Based on Expected Product Liking (EF4)

Three clusters were retained, and only in Cluster 1, the smallest of these clusters (13%), did flavour preference override preference for product type (dairy vs. PBCCA). This was seen in Table 2 from the positive B-W scores in Cluster 1 for products with original/plain flavour and negative B-W scores for products with any other flavour (see also Part SM12 in the Supplementary Materials for a plot of mean B-W scores by cluster). Among the five products with original/plain flavours, the PBCCAs had expected liking scores that statistically were the same as those observed for low-fat and lactose-free dairy variants, except for the PBCCA made with coconut oil, which was expected to be less liked than the dairy low-fat variant.

Clusters 2 and 3 had very similar profiles for expected product liking except for the salmon-flavoured cream cheese, which was polarising, with a low expected liking in Cluster 2 and a high expected liking in Cluster 3. The correlation coefficient between the two sets of B-W scores was 0.98 when the salmon-flavoured sample was excluded (Part SM12 in the Supplementary Materials). Regarding the PBCCA samples, negative expectations were consistently observed. These samples were always expected to be significantly less liked than the cream cheese variants, regardless of whether they were regular, low-fat, or lactose-free. In these clusters, consumers did not expect to like the original/plain PBCCAs made from different PB ingredients differently.

### 3.4. Product Choice (EF5)

In a hypothetical choice situation where participants could select between a product (any flavour among those evaluated in the study) to take home free of charge, PBCCAs were selected by 11% of participants (Table 1). The strongest predictor was stated liking for PBCCA followed by stated PBCCA consumption frequency (Table 4). Correspondingly, liking and more frequent consumption of cream cheese were negative predictors for PBCCA product choice. Relative to participants who identified as vegetarian, those who identified as following an omnivore dietary style were less likely to select PBCCA in the hypothetical take home task.

## 4. Discussion

### 4.1. Evidence of Systematic Negative PBCCA Expectations

Fitting with expectations, systematic negative expectations for PBCCAs were found (EF1). These findings align with those in the existing literature, indicating that PB food alternatives are often met with lower expectations than traditional products (dairy, meat, and seafood), largely because of anticipated differences in sensory quality [32,34,68,69].

As is consistent with previous research involving the sensory evaluation of PBCCA and dairy cream cheeses, dairy cream cheeses in this study were more associated with positive emotions such as ‘happy/satisfied’ and ‘secure/at ease’, while PBCCAs were linked to more negative emotions like ‘unhappy/dissatisfied’ and ‘dull/bored’ [30]. These emotional associations further support EF1 that negative expectations for PBCCAs would persist across flavour variants, highlighting a systematic bias favouring dairy-based products among the participating consumers. Such biases may be driven by familiarity with traditional dairy textures and flavours and perceptions that PB alternative foods and ingredients are artificial or lack authenticity [27,70]. Similar consumer concerns exist for PB alternatives to meat [71].

The observed influences of sensory drivers on expected product liking (EF2) highlighted the critical role of sensory and non-sensory attributes in consumer acceptance of dairy and dairy-alternative products. Research has shown that sensory characteristics such as creaminess, smoothness, and flavour intensity are primary drivers of liking for dairy and PB cream cheese, as these attributes align with consumers’ expectations for dairy-based products [30,33,72,73]. Negative sensory associations, such as ‘nutty flavour’ and ‘oat/grain flavours’, which deviated from typical cream cheese profiles, led to a hedonic penalty, underscoring findings by Jaeger et al. [30] that PB flavours unfamiliar to traditional dairy consumers may reduce product liking. This offers clear direction to product developers and marketers to seek product formulations that minimise characteristics that penalise positive product responses while emphasising desired characteristics on packaging and in promotions. For example, packaging could emphasise that the product has a creamy and smooth texture and reduce emphasis on the PB ingredients used in the product.

The observed influences of non-sensory drivers on expected product liking (EF2) were also consistent with those in the existing literature. Emotional responses were shown to significantly affect consumer expectations and product liking, with positive emotions like ‘happy/satisfied’ and ‘relaxed/calm’ leading to a higher expected liking in line with prior research on food products [74,75,76]. Similarly, negative emotions such as ‘unhappy/dissatisfied’ were linked to reduced liking, as found in prior research with cream cheese and PBCCAs [30,77]. Conceptual associations, particularly those related to tradition, authenticity, and naturalness, also play a substantial role in consumer expectations of dairy and PB dairy alternatives, potentially because consumers might associate these conceptualisations with higher product quality and trustworthiness [31,37]. This is consistent with the findings in this study, where terms like ‘wholesome’ and ‘trustworthy’ led to higher expected liking, while negative associations like ‘artificial’ and ‘unfamiliar’ diminished it. Tentatively, marketers can draw on positive brand equity to seek transference of trustworthiness to PB product alternatives. Similarly, they may be able to harness perceptions regarding the healthiness and naturalness of the PB ingredients (e.g., almonds, oats, coconut oil) to boost product perceptions. Collectively, the EF2 results align with those in the broader body of literature, underscoring the importance of aligning sensory and non-sensory product attributes to meet consumer expectations for traditional dairy products when developing PB dairy alternatives [17,34,37]. Similar findings exist for PB meat alternatives [78].

A strong correlation between expected liking and expected versatility (EF3) supports prior research demonstrating that versatile products are often rated more favourably by consumers, as versatility suggests adaptability across a range for which a product can be used [40,79]. Previous sensory research has established that dairy cream cheese is generally perceived as more versatile than PBCCAs, although it also indicated that both dairy cream cheeses and PBCCAs that were perceived as mild or bland were less liked by consumers [30]. These findings correspond with the results of this study, where mild or bland flavours emerged as negative drivers of expected liking but did not significantly affect expected versatility. Conversely, garlic and herb flavours positively influenced expected liking yet similarly showed no significant effect on versatility. This suggests that these flavours can enhance consumer appeal in cream cheese applications without necessarily influencing perceptions of versatility. The potential of adding flavours to increase the acceptability of PB products was also found in other products, such as plant-based milk alternatives [80]. Furthermore, a strong alignment was observed between expected liking and expected versatility across most conceptual attributes, except for health, which served as a driver for expected versatility but did not significantly contribute to expected liking. A tentative explanation is that consumers may hold an implicit belief that unhealthy foods taste better than healthy foods [81,82]. Conversely, social norms regarding serving healthy foods [83,84] could contribute to increased perceived versatility. For product developers and marketers, an implication of the EF4 findings is to find ways to increase the perceived versatility of PBCCAs in a manner that does not compromise expected liking. Success herein should increase consumer appeal.

Three clusters were identified, supporting the existence of distinct consumer groups with varying flavour and product type preferences within the cream cheese category (EF4). The smallest of the clusters (Cluster 1, 13%) was the only group where flavour preference preceded product type preference (dairy vs. PBCCA). In this cluster, products with original/plain flavours—whether dairy or PB—received positive scores for expected liking. This indicates that for a small subset of consumers, flavour characteristics can surpass the preference for traditional dairy, aligning with the literature that suggests certain consumer segments are more open to PB alternatives [20,85]. However, PBCCAs made with coconut oil were an exception, scoring lower than the low-fat dairy variant in Cluster 1, suggesting that even in flavour-focused segments, specific PB ingredients may pose barriers to acceptance. It remains uncertain whether these specific expectations for PB products are grounded in actual sensory experiences, particularly given prior findings of lower acceptance for coconut-based cheese alternatives than dairy-based options [30,86]. Sensory research on yoghurt products has demonstrated similar acceptance scores between certain dairy-based and coconut-based PB alternatives [87,88]. This suggests a need to investigate whether these PB expectations vary by food categories and to what extent they are influenced by prior sensory experiences. Such exploration could provide insights into the generalisability of sensory biases toward PB alternatives across different food categories.

While Clusters 2 and 3 exhibited a stronger preference for dairy-based products, the salmon-flavoured cream cheese was polarising between these two clusters, indicating the potential for flavour-specific segmentation within dairy options. For PBCCAs, there was no significant differentiation in expected liking based on PB ingredients (e.g., almond/oat vs. coconut oil), suggesting that the PB nature itself, rather than specific ingredients, drives lower expectations in these two clusters [33]. This outcome highlights the importance of addressing both flavour and ingredient perception when developing PB alternatives, as consumers in more traditional clusters may be less receptive regardless of flavour [1]. Furthermore, it showed the need for targeted marketing strategies to appeal to both dairy-oriented and flavour-flexible segments. For product developers, the current results also imply that there is likely value in exploring the appeal of more diverse PBBCA flavour options.

The hypothetical choice task results confirm a strong behavioural preference for traditional cream cheese over PBCCAs (EF5), with only 11% of participants opting for PBCCAs. Positive prior experiences, such as high stated liking and frequent PBCCA consumption, were the strongest predictors of PBCCA selection, while a preference for dairy cream cheese and frequent dairy consumption reduced the likelihood of choosing plant-based alternatives. Additionally, vegetarians showed greater openness to PBCCAs than omnivores, consistent with research indicating that dietary lifestyle influences acceptance of PB alternative products [18,89]. These findings align with those of prior studies, suggesting that familiarity may mitigate sensory biases against PB food alternatives (dairy and beyond) [80,90] and that targeted strategies to improve sensory appeal and align PBCCAs with traditional dairy expectations may broaden their acceptance among omnivores [17,34,91].

### 4.2. Limitations and Suggestions for Future Research

The research set out to perform a category appraisal study but did not include all commercially available variants due to the excessive burden this would have placed on participants. This may limit the generalisability of the findings. However, care was taken to include stimuli representing market leaders and niche products concerning flavour and excluded ingredients. Regarding flavour, care was also taken to include both savoury and sweet variants.

The richness of the obtained data creates opportunities for further analyses, including establishing the sensory drivers of non-sensory product perceptions. It is beyond the scope of the present research to do so, but the expectation, based on such analyses performed for PB alternatives to yoghurt [31,58], is that sensory attributes that positively impact mean expected liking will also contribute to increased emotions with positive valence (vice versa for products that elicited emotions with negative valence).

The negative sensory expectations observed in this study were based on written descriptions rather than actual product experience, and consequently the results may not fully align with consumers’ sensory perceptions upon tasting. Future work that involves actual tasting is recommended. However, it is important to emphasise that the current findings are consistent with previous studies in which consumers participated in sensory evaluations of PB cream cheese alternatives (PBCCAs), where similar negative biases were observed [30,33]. This alignment suggests that, despite the absence of direct sensory exposure in this study, the written information effectively invoked comparable expectations to those formed during actual product interactions. This point was also recently made by Jaeger et al. [58] based on consumer research with PB alternatives to yoghurt.

It would be relevant to extend the research to product types beyond cream cheese to understand how generalisable the present results are. A first step would be other types of cheese. Cold-sliced meats and their PB options could also be an interesting case study for a category appraisal approach. Other extensions should consider consumers in other countries and more formal comparisons of consumers with different dietary habits.

Vegans were excluded from the present research. This was meaningful in light of the implemented category appraisal approach since vegans would not consume dairy cream cheese. This exclusion does not imply that consumer insights from vegans lack relevance. However, vegan consumers already follow a more sustainable diet. To achieve a major impact and food system transformation, it is consumers who still rely on meat and dairy (i.e., omnivore or flexitarian diet) (Table 1) that need to change their diets. They were the focus of the present research.

## 5. Conclusions

The present research observed negative consumer expectations for plant-based alternatives to cream cheese (PBCCA) relative to their dairy-based counterparts. This finding, obtained in a sample of omnivore and flexitarian UK consumers, extended past research by showing that negative product expectations transcend flavour variants. The category appraisal approach, novel to product-focused consumer expectation research, also revealed that PBCCA product expectations were more negative, on average, than expectations for other product variants in the cream cheese category, such as those that are low-fat or lactose-free.

The observed negative expectations were systemic and encompassed expected sensory, emotional, conceptual, and versatility product associations. The analyses identified a diverse range of drivers of expected liking and versatility, including soft texture and creamy/smooth mouthfeel (positive impacts) and strong flavours linked to plant-based ingredients (negative impacts). Lifts were also associated with positive and activated emotions. These were among the insights that can guide product developers, who, in concert with marketers, should also strive to diminish perceptions that PBCCAs are artificial and unfamiliar. The results from the cluster analysis also indicated that product promotions must be tailored to different consumer segments.

In a hypothetical choice situation, only 11% of participants indicated that they would choose a PBCCA product in favour of a dairy cream cheese. This correlated with the systematic evidence of negative PBCCA expectations. In turn, this feeds into a needed rethinking of the appeal of this category and its relevance for improving sustainability in food consumption.

## Figures and Tables

**Table 1:** Summary participant characteristics in (a) sample of 1093 adult United Kingdom participants, (b) Cluster 1 (*n* = 126), (c) Cluster 2 (*n* = 530), and (d) Cluster 3 (*n* = 291). Values are expressed as percentages (%) of the sample unless otherwise stated.

Participant Characteristics	Total Sample *n* = 1093	Cluster 1 *n* = 126	Cluster 2 *n* = 530	Cluster 3 *n* = 291
Gender				
Men	47	40	43	51
Women	53	60	57	49
Other	<1	0	<1	<1
Age group				
18–45 years old	46	41	48	35
46–69 years old	54	59	52	65
Annual household income before tax				
Less than GBP 35,000	40	44	39	40
GBP 35,000 or more	56	52	57	56
Prefer not to answer	4	4	4	4
Educational attainment				
Below college	40	40	39	47
College or higher	60	59	60	53
Other/Prefer not to answer	<1	2	<1	0
Dietary habits ^#^				
Omnivore	72	63	70	78
Flexitarian	23	28	22	21
Vegetarian	6	9	8	1
Stated liking ^1^				
Cream cheese (dairy)	7.6 (1.3)	7.6 (1.3)	7.6 (1.2)	7.7 (1.3)
Plant-based alternative to cream cheese (non-dairy)	4.9 (2.0)	5.5 (1.8)	4.8 (1.9)	4.3 (1.9)
Consumption frequency ^2^				
Cream cheese (dairy)	5.2 (1.6)	5.0 (1.6)	5.1 (1.5)	5.2 (1.6)
Plant-based alternative to cream cheese (non-dairy)	2.3 (1.9)	2.4 (2.0)	2.0 (1.7)	2.0 (1.7)
Cream cheese choice to take home				
Dairy version (any flavour of your choice)	89	82	91	97
Plant-based alternative (any flavour of your choice)	11	18	9	3

Notes. (%) After rounding, some percentage values may not add to 100. (#) Omnivores defined as those who do not follow any limitation concerning consumption of meat and fish; flexitarians defined as those who consciously consume limited quantity of either all types or specific types of meat; vegetarians defined as people who totally limit consumption of meat and fish. (^1^) Stated liking measured on 9-point hedonic scale from 1 = ‘dislike extremely’ to 9 = ‘like extremely’. Values expressed as mean and standard deviation. (^2^) Consumption frequency measured on 9-point scale with categories: 1 = ‘Never or less than once a year’; 2 = ‘1–3 times a year’; 3 = ‘Every 2–3 months’; 4 = ‘Once a month’; 5 = ‘2–3 times a month’; 6 = ‘Once a week’; 7 = ‘2–4 times a week’; 8 = ‘5–6 times a week’; and 9 = ‘Once daily or more often’. Values expressed as mean and standard deviation.

**Table 2:** Written product stimuli (*n* = 13) as used in study. Full descriptions are shown as presented to consumers. Six products marked by * comprised subset of stimuli for which expected sensory product characteristics and conceptual associations were measured. Results for EF1 (based on responses from 1093 participants) are as follows: (i) average expected product liking as B-W (best-minus-worst) scores (+4 to −4) ^$^ and (ii) average expected product versatility (1 = ‘low versatility of use/few different uses’; 7 = ‘high versatility of use/many different uses’). Results for EF4 (based on responses from 947 participants) are expected product liking as B-W scores (+4 to −4) ^$^ by cluster.

Product Descriptions	Short Name in Figures and Tables	Aggregate Expected Product Liking ^$$^ (EF1)	Aggregate Expected Product Versatility ^$$^ (EF1)	Expected Product Liking in Cluster 1 ^$$$^ (13%) (EF4)	Expected Product Liking in Cluster 2 ^$$$^ (56%) (EF4)	Expected Product Liking in Cluster 3 ^$$$^ (31%) (EF4)
Cream cheese, original/plain flavour *	Orig	1.99 (B)	6.18 (A)	3.2 (A)	2.2 (C)	2.0 (B)
Cream cheese, original/plain flavour, plant-based alternative (almonds and oats) *	Orig_PB_AO	−0.51 (G)	4.63 (DE)	1.3 (BC)	−0.7 (G)	−1.2 (F)
Cream cheese, original plain flavour, plant-based alternative (coconut oil)	Orig_PB_COC	−0.76 (G)	4.53 (EF)	1.0 (C)	−0.9 (G)	−1.4 (F)
Cream cheese, original/plain flavour, low-fat *	Orig_LowFat	0.98 (D)	5.71 (B)	1.8 (B)	1.4 (D)	0.6 (C)
Cream cheese, original/plain flavour, lactose-free	Orig_LactFr	0.10 (EF)	5.18 (C)	1.4 (BC)	0.4 (F)	−0.5 (E)
Cream cheese, garlic and herbs flavour *	G&H	2.31 (A)	5.51 (B)	−0.4 (D)	3.1 (A)	3.0 (A)
Cream cheese, garlic and herbs flavour, plant-based alternative (almonds and oats) *	G&H_PB_AO	−0.04 (F)	4.38 (F)	−0.5 (D)	0.2 (F)	−0.4 (E)
Cream cheese, garlic and herbs flavour, low-fat *	G&H_LowFat	1.65 (C)	5.21 (C)	−0.7 (DE)	2.5 (B)	2.0 (B)
Cream cheese, garlic and herbs flavour, lactose-free	G&H_LactFr	0.27 (E)	4.81 (D)	−1.1 (DEF)	0.9 (E)	0.1 (D)
Cream cheese, strawberry flavour	Strawb	−1.53 (H)	2.60 (H)	−1.3 (EF)	−2.1 (H)	−1.8 (G)
Cream cheese, strawberry flavour, plant-based alternative (almonds and oats)	Strawb_PB_AO	−1.99 (I)	2.67 (H)	−1.1 (DEF)	−2.5 (I)	−2.6 (H)
Cream cheese, milk chocolate flavour	MlkChoc	−1.74 (HI)	2.59 (H)	−1.4 (F)	−2.1 (H)	−2.4 (H)
Cream cheese, salmon flavour	Salmon	−0.75 (G)	3.52 (G)	−2.2 (G)	−2.4 (HI)	2.7 (A)

Notes. (^$^) B-W scores with positive values indicate that product liking is higher than average across all products, while the negative B-W scores indicate the reverse. The more positive the B-W values, the more the product is expected to be liked. The more negative the B-W score is, the less the product is expected to be liked. (^$$^) Standard errors around product averages were SE = 0.05 for expected liking (all products) and SE = 0.05 for expected versatility (all products). Tukey’s HSD post hoc test results are shown in brackets. Within columns, products with different letters had significantly different averages at the 5% level of significance. (^$$$^) Standard errors around expected product liking averages were SE = 0.14 for Cluster 1 (all products); SE = 0.05 for Cluster 2 (all products); and SE = 0.08 for Cluster 3 (all products). Tukey’s HSD post hoc test results are shown in brackets. Within columns, products with different letters had significantly different averages at the 5% level of significance.

**Table 3:** Results for EF2. Term citation frequencies (sensory, emotional, conceptual) and mean impact ^$^ on expected cream cheese product liking ^$$^ and expected product versatility ^$$$^ following penalty/lift analysis performed on 6-sample stimuli subset ^$$$$^.

CATA Term	Term Citation Frequency (%) ^$$$$$^	Mean Impact on Expected Liking	Mean Impact on Expected Versatility
**Expected sensory product characteristics**			
Buttery flavour	10.6	0.6 **	0.6 ***
Cow-like flavour	5.1 ^#^	0.1	0.3
Creamy/smooth mouthfeel	42.9	1.2 ***	1.0 ***
Dense texture	9.7	−0.2	0.0
Dissolves quickly in mouth	11.2	0.6 **	0.6 ***
Firm	6.8 ^#^	−0.1	0.3
Garlic flavour	32.7	0.6 ***	−0.2
Herbs flavour	32.8	0.6 ***	−0.1
Light/airy texture	19.2	0.5 **	0.6 ***
Mild/bland flavour	18.6	−0.8 ***	−0.1
Nutty flavour	9.1	−1.4 ***	−0.7 ***
Oat/grain flavour	9.8	−1.4 ***	−0.9 ***
Salty	11.2	0.3	0.1
Savoury flavour	31.2	0.9 ***	0.4 **
Shiny/glossy appearance	10.8	0.8 ***	0.7 ***
Soft	48.9	1.0 ***	0.8 ***
Sour/tangy	8.9 ^#^	−0.3	−0.3 *
Sticky mouthfeel	5.6 ^#^	−1.3 ***	−0.9 ***
Strong/intense flavour	18.4	0.7 ***	0.3 *
Sweet	3.4 ^#^	−0.9 ***	0.4
**Expected emotional product associations**			
Energetic, Excited	10.2	0.9 ***	1.0 ***
Enthusiastic, Inspired	12.7	1.2 ***	0.7 ***
Happy, Satisfied	38.6	1.6 ***	1.1 ***
Secure, At Ease	21.6	0.9 ***	0.8 ***
Relaxed, Calm	32.4	0.9 ***	0.8 ***
Passive, Quiet	12.1	−0.2	0.1
Dull, Bored	9.7	−1.4 ***	−0.8 ***
Blue, Uninspired	5.4 ^#^	−1.7 ***	−1.2 ***
Unhappy, Dissatisfied	10.0	−2.5 ***	−2.0 ***
Tense, Bothered	3.9 ^#^	−1.8 ***	−1.8 ***
Jittery, Nervous	3.8 ^#^	−2.4 ***	−1.5 ***
Active, Alert	8.8 ^#^	0.3	0.4 **
**Expected conceptual product associations**			
Artificial	8.0 ^#^	−1.5 ***	−1.5 ***
Artisanal	5.5 ^#^	0.3	−0.1
Cheap	5.3 ^#^	−0.3	0.0
Comforting	25.4	1.6 ***	0.9 ***
Ethical	6.9 ^#^	−0.2	0.5 **
Genuine	18.8	1.0 ***	0.8 ***
Healthy	28.4	0.1	0.6 ***
Natural	30.6	0.6 ***	0.7 ***
Nutritious	21.0	0.6 ***	0.7 ***
Respectful	6.2 ^#^	0.2	0.5 **
Simple	33.4	0.6 ***	0.8 ***
Sophisticated	5.9 ^#^	0.7 **	0.7 **
Traditional	22.8	1.2 ***	0.8 ***
Trustworthy	20.1	0.9 ***	1.0 ***
Unfamiliar	10.8	−1.9 ***	−1.6 ***
Unnecessary	8.6 ^#^	−2.0 ***	−2.0 ***
Versatile	24.4	1.0 ***	1.1 ***
Wholesome	18.6	0.9 ***	0.7 ***

Notes. CATA = Check-all-that-apply. (^$^) Asterisks indicate if mean impact is significantly different from zero as follows: * if *p* < 0.05, ** if *p* < 0.01, and *** if *p* < 0.001. (^$$^) Measured as B-W scores (+4 to −4). (^$$$^) Measured on 7-point scale (1 = ‘low versatility of use/few different uses’; 7 = ‘high versatility of use/many different uses’). (^$$$$^) Six samples were as follows: cream cheese, garlic and herbs flavour, low-fat; cream cheese, garlic and herbs flavour, plant-based alternative (almonds and oats); cream cheese, garlic and herbs flavour; cream cheese, original/plain flavour, low-fat; cream cheese, original/plain flavour, plant-based alternative (almonds and oats); cream cheese, original/plain flavour. (^$$$$$^) When 100 or fewer consumers have used a CATA term (9.15%), indicated as # and serves as signal to be cautious when interpreting test results.

**Table 4:** Results for EF5. Model parameters from logistic regression to predict choice of take-home cream cheese product (any flavour variant) based on expected liking, frequency of consumption, and stated dietary preference. PBCCA = plant-based alternatives to cream cheese.

Source	Standardised Coefficient	Standard Error	Wald χ2	Pr > χ2	Odds Ratio (OR)	95% OR Confidence Interval
Stated liking: Cream cheese	−0.29	0.07	18.98	<0.0001	0.66	0.54–0.79
Stated liking: PBCCA	0.73	0.10	48.51	<0.0001	1.95	1.62–2.35
Stated consumption frequency: Cream cheese	−0.23	0.08	8.97	0.003	0.77	0.64–0.91
Stated consumption frequency: PBCCA	0.37	0.08	23.31	<0.0001	1.42	1.23–1.64
Dietary habit: Omnivore	−0.26	0.10	7.14	0.01	0.35	0.17–0.76
Dietary habit: Flexitarian	−0.02	0.09	0.06	0.81	0.91	0.42–1.98
Dietary habit: Vegetarian	0.00	0.00				

Notes. Model estimation: df = 1086; −2Log(likelihood) = 517.5; Cox and Snell R^2^ = 0.21.

## Data Availability

The original contributions presented in the study are included in the article/Supplementary Material, further inquiries can be directed to the corresponding author.

## Supplementary Materials

The following supporting information can be downloaded at: https://www.mdpi.com/article/10.3390/foods14030445/s1. PART SM1. Supporting table with sample of 1093 participants and UK 2021 Census comparative data for selected participant characteristics; PART SM2. A screenshot from the BWS task used in the online survey to elicit the expected liking for cream cheese and its PB alternatives, and BIBD defining the combination of products in each BWS choice set; PART SM3. Number of participants per product stimuli in six-sample subset; PART SM4. Data quality statement; PART SM5. ErrVarNorm (EVN) data quality index for BWS task (sustainable food choice motives); PART SM6. Illustration of how inconsistent responding in Case 1 BWS task influences ErrVarNorm (EVN) index; PART SM7. Dendrogram following hierarchical cluster analysis on B-W scores for expected liking of evaluated product stimuli (EF4). Comparison of solutions with 2, 3, and 4 clusters led to final solution with 3 clusters (here shown as C1, C2, C3) based on dissimilarity criterion. Horizontal line identifies which participants are allocated to which cluster; PART SM8. Results for EF1. Supplement to Figure 2A with labelling of all products and terms; PART SM9. Results for EF1. Two-dimensional solutions following Correspondence Analysis based on expected sensory associations to 6 stimuli in product subset. (A) Biplot showing samples and terms. (B) Product space with 95% confidence ellipses around average variable positions; PART SM10. Results for EF1. Two-dimensional solutions following Correspondence Analysis based on expected emotional associations to 6 stimuli in product subset. (A) Biplot showing samples and terms. (B) Product space with 95% confidence ellipses around average variable positions; PART SM11. Results for EF1. Two-dimensional solutions following Correspondence Analysis based on expected conceptual associations to 6 stimuli in product subset. (A) Biplot showing samples and terms. (B) Product space with 95% confidence ellipses around average variable positions; PART SM12. Results for EF4. (A) Profile plot of mean expected product liking for 13 product stimuli included in research. Shown as B-W scores (+4 to −4) by cluster based on responses from 947 participants. (B) Scatterplot of B-W scores for 12 product stimuli in Cluster 2 and Cluster 3 (salmon-flavoured sample excluded).

## References

[1] Aschemann-Witzel J., Gantriis R.F., Fraga P., Perez-Cueto F.J. (2021). Plant-based food and protein trend from a business perspective: Markets, consumers, and the challenges and opportunities in the future. Crit. Rev. Food Sci. Nutr..

[2] Boukid F. (2021). Plant-based meat analogues: From niche to mainstream. Eur. Food Res. Technol..

[3] Bonetti G.G., van Hooven C., Onorati M.G. (2024). Planting Seeds of Change in Foodstyles: Growing Brand Strategies to Foster Plant-Based Alternatives Through Online Platforms. Gastronomy.

[4] Gilbert N. (2012). One-third of our greenhouse gas emissions come from agriculture. Nature.

[5] Godfray H.C.J., Beddington J.R., Crute I.R., Haddad L., Lawrence D., Muir J.F., Pretty J., Robinson S., Thomas S.M., Toulmin C. (2010). Food security: The challenge of feeding 9 billion people. Science.

[6] Poore J., Nemecek T. (2018). Reducing food’s environmental impacts through producers and consumers. Science.

[7] Marlow H.J., Hayes W.K., Soret S., Carter R.L., Schwab E.R., Sabate J. (2009). Diet and the environment: Does what you eat matter?. Am. J. Clin. Nutr..

[8] Sabaté J., Soret S. (2014). Sustainability of plant-based diets: Back to the future. Am. J. Clin. Nutr..

[9] Bryant C.J. (2022). Plant-based animal product alternatives are healthier and more environmentally sustainable than animal products. Future Foods.

[10] Fehér A., Gazdecki M., Véha M., Szakály M., Szakály Z. (2020). A Comprehensive Review of the Benefits of and the Barriers to the Switch to a Plant-Based Diet. Sustainability.

[11] Satija A., Bhupathiraju S.N., Rimm E.B., Spiegelman D., Chiuve S.E., Borgi L., Willett W.C., Manson J.E., Sun Q., Hu F.B. (2016). Plant-based dietary patterns and incidence of type 2 diabetes in US men and women: Results from three prospective cohort studies. PLoS Med..

[12] Curtain F., Grafenauer S. (2019). Plant-based meat substitutes in the flexitarian age: An audit of products on supermarket shelves. Nutrients.

[13] Good Food Institute US Retail Market Insights Plant-Based Foods. https://gfi.org/marketresearch/.

[14] Cardello A.V., MacFie H. (2007). Measuring consumer expectations to improve food product development. Consumer-Led Food Product Development.

[15] Piqueras-Fiszman B., Spence C. (2015). Sensory expectations based on product-extrinsic food cues: An interdisciplinary review of the empirical evidence and theoretical accounts. Food Qual. Prefer..

[16] Michel F., Hartmann C., Siegrist M. (2021). Consumers’ associations, perceptions and acceptance of meat and plant-based meat alternatives. Food Qual. Prefer..

[17] Giacalone D., Clausen M.P., Jaeger S.R. (2022). Understanding barriers to consumption of plant-based foods and beverages: Insights from sensory and consumer science. Curr. Opin. Food Sci..

[18] Perez-Cueto F.J., Rini L., Faber I., Rasmussen M.A., Bechtold K.-B., Schouteten J.J., De Steur H. (2022). How barriers towards plant-based food consumption differ according to dietary lifestyle: Findings from a consumer survey in 10 EU countries. Int. J. Gastron. Food Sci..

[19] Onwezen M.C., Verain M.C., Dagevos H. (2022). Positive emotions explain increased intention to consume five types of alternative proteins. Food Qual. Prefer..

[20] Cardello A.V., Llobell F., Giacalone D., Chheang S.L., Jaeger S.R. (2022). Consumer preference segments for plant-based foods: The role of product category. Foods.

[21] Singh M., Trivedi N., Enamala M.K., Kuppam C., Parikh P., Nikolova M.P., Chavali M. (2021). Plant-based meat analogue (PBMA) as a sustainable food: A concise review. Eur. Food Res. Technol..

[22] Szenderak J., Frona D., Rakos M. (2022). Consumer acceptance of plant-based meat substitutes: A narrative review. Foods.

[23] Zahari I., Östbring K., Purhagen J.K., Rayner M. (2022). Plant-based meat analogues from alternative protein: A systematic literature review. Foods.

[24] Andreani G., Sogari G., Marti A., Froldi F., Dagevos H., Martini D. (2023). Plant-based meat alternatives: Technological, nutritional, environmental, market, and social challenges and opportunities. Nutrients.

[25] De Angelis D., van der Goot A.J., Pasqualone A., Summo C. (2024). Advancements in texturization processes for the development of plant-based meat analogs: A review. Curr. Opin. Food Sci..

[26] Mekanna A.N., Issa A., Bogueva D., Bou-Mitri C. (2024). Consumer perception of plant-based milk alternatives: Systematic review. Int. J. Food Sci. Technol..

[27] Sethi S., Tyagi S.K., Anurag R.K. (2016). Plant-based milk alternatives an emerging segment of functional beverages: A review. J. Food Sci. Technol..

[28] Oyeyinka A.T., Odukoya J.O., Adebayo Y.S. (2019). Nutritional composition and consumer acceptability of cheese analog from soy and cashew nut milk. J. Food Process. Preserv..

[29] Appiani M., Cattaneo C., Laureati M. (2025). Plant-based fish analogues vs. fish: Assessment of consumer perception, acceptance, and attitudes. Food Qual. Prefer..

[30] Jaeger S.R., Jin D., Roigard C.M. (2024). Plant-Based Alternatives Need Not Be Inferior: Findings from a Sensory and Consumer Research Case Study with Cream Cheese. Foods.

[31] Cardello A.V., Llobell F., Jin D., Ryan G.S., Jaeger S.R. (2024). Sensory drivers of liking, emotions, conceptual and sustainability concepts in plant-based and dairy yoghurts. Food Qual. Prefer..

[32] Collier E.S., Harris K.L., Bendtsen M., Norman C., Niimi J. (2023). Just a matter of taste? Understanding rationalizations for dairy consumption and their associations with sensory expectations of plant-based milk alternatives. Food Qual. Prefer..

[33] Waehrens S.S., Faber I., Gunn L., Buldo P., Frøst M.B., Perez-Cueto F.J. (2023). Consumers’ sensory-based cognitions of currently available and ideal plant-based food alternatives: A survey in Western, Central and Northern Europe. Food Qual. Prefer..

[34] Greis M., Nolden A.A., Kinchla A.J., Puputti S., Seppä L., Sandell M. (2023). What if plant-based yogurts were like dairy yogurts? Texture perception and liking of plant-based yogurts among US and Finnish consumers. Food Qual. Prefer..

[35] Heussen F., Holthuysen N., Kremer S., Rason J., Worch T. (2023). Beyond liking: Innovative approach using CATA to better understand consumer’s associations to products. Food Qual. Prefer..

[36] Thomson D.M., Coates T., Meiselman H. (2021). Concept profiling—Navigating beyond liking. Emotion Measurement.

[37] Panagiotou M., Kaloudis E., Koukoumaki D.I., Bountziouka V., Giannakou E., Pandi M., Gkatzionis K. (2024). Key Drivers of Consumption, Conceptual, Sensory, and Emotional Profiling of Cheeses Based on Origin and Consumer Familiarity: A Case Study of Local and Imported Cheeses in Greece. Gastronomy.

[38] Jaeger S.R., Roigard C.M., Le Blond M., Hedderley D.I., Giacalone D. (2019). Perceived situational appropriateness for foods and beverages: Consumer segmentation and relationship with stated liking. Food Qual. Prefer..

[39] Jaeger S.R., Roigard C.M., Ryan G., Jin D., Giacalone D. (2021). Consumer segmentation based on situational appropriateness ratings: Partial replication and extension. Food Qual. Prefer..

[40] Cardello A.V., Llobell F., Giacalone D., Roigard C.M., Jaeger S.R. (2022). Plant-based alternatives vs dairy milk: Consumer segments and their sensory, emotional, cognitive and situational use responses to tasted products. Food Qual. Prefer..

[41] Giacalone D. (2018). Sensory and consumer approaches for targeted product development in the agro-food sector. Case Stud. Tradit. Food Sect..

[42] International Organization for Standardization Market, Opinion and Social Research, Including Insights and Data Analytics—Vocabulary and Service Requirements (ISO Standard No. 20252). https://www.iso.org/obp/ui/#iso:std:iso:20252:ed-3:v1:en.

[43] Innova Market Insights Cheese Trends: Global Market Overview. https://www.innovamarketinsights.com/trends/cheese-trends/.

[44] Statista Global Cheese Market—Statistics & Facts. https://www.statista.com/topics/6586/global-cheese-market/#topicOverview.

[45] OECD/FAO (2020). OECD-FAO Agricultural Outlook 2020–2029.

[46] Statista Global per Capita Consumption of Cheese 2023, by Country. https://www.statista.com/statistics/527195/consumption-of-cheese-per-capita-worldwide-country/.

[47] Fox P.F., McSweeney P.L., McSweeney P.L.H., Fox P.F., Cotter P.D., Everett D.W. (2017). Chapter 1—Cheese: An overview. Cheese: Chemistry, Physics and Microbiology.

[48] Future Market Insights Inc. Cream Cheese Market Outlook (2023 to 2033). https://www.futuremarketinsights.com/reports/cream-cheese-market.

[49] Philadelphia Our Products. https://www.creamcheese.com/products.

[50] Pombo A.F.W. (2021). Cream cheese: Historical, manufacturing, and physico-chemical aspects. Int. Dairy J..

[51] Foguel A., Neves Rodrigues Ract J., Claro da Silva R. (2021). Sensory characterization of commercial cream cheese by the consumer using check-all-that-apply questions. J. Sens. Stud..

[52] Craig W.J., Mangels A.R., Brothers C.J. (2022). Nutritional profiles of non-dairy plant-based cheese alternatives. Nutrients.

[53] Louviere J.J., Flynn T.N., Marley A.A.J. (2015). Best-Worst Scaling: Theory, Methods and Applications.

[54] Cochran W.G., Cox G.M. (1957). Experimental Design.

[55] Ares G., Jaeger S., Delarue J., Lawlor J.B. (2023). Check-all-that-apply (CATA) questions with consumers in practice: Experimental considerations and impact on outcome. Rapid Sensory Profiling Techniques.

[56] Jaeger S.R., Roigard C.M., Jin D., Xia Y., Zhong F., Hedderley D.I. (2020). A single-response emotion word questionnaire for measuring product-related emotional associations inspired by a circumplex model of core affect: Method characterisation with an applied focus. Food Qual. Prefer..

[57] Thomson D.M.H., Meiselman H. (2016). Conceptual profiling. Emotion Measurement.

[58] Jaeger S., Chheang S., Llobell F., Cardello A. (2024). Consumers’ Expectations of Liking, Emotional, Conceptual and Sustainability Characteristics of Dairy, Plant-Based and Sustainable Yoghurts. J. Sens. Stud..

[59] De Backer C.J., Hudders L. (2015). Meat morals: Relationship between meat consumption consumer attitudes towards human and animal welfare and moral behavior. Meat Sci..

[60] Jaeger S.R., Cardello A.V. (2022). Factors affecting data quality of online questionnaires: Issues and metrics for sensory and consumer research. Food Qual. Prefer..

[61] Newman A., Bavik Y.L., Mount M., Shao B. (2021). Data collection via online platforms: Challenges and recommendations for future research. Appl. Psychol..

[62] Schonlau M., Toepoel V. (2015). Straightlining in Web survey panels over time. Surv. Res. Methods.

[63] Llobell F., Choisy P., Chheang S.L., Jaeger S.R. (2024). Measurement and evaluation of participant response consistency in Case 1 Best-Worst-Scaling (BWS) in food consumer science. Food Qual. Prefer..

[64] Marley A.A., Louviere J.J. (2005). Some probabilistic models of best, worst, and best–worst choices. J. Math. Psychol..

[65] Meyners M., Castura J.C., Carr B.T. (2013). Existing and new approaches for the analysis of CATA data. Food Qual. Prefer..

[66] Hasted A. Understanding Consumers by Clustering—Successes, Problems and Pitfalls. https://www.sensometrics2020.com/_files/ugd/97f264_785a48a2487a424d9ce7c09c7e991e90.pdf.

[67] Lumivero (2024). XLSTAT Statistical and Data Analysis Solution.

[68] Ettinger L., Falkeisen A., Knowles S., Gorman M., Barker S., Moss R., McSweeney M.B. (2022). Consumer perception and acceptability of plant-based alternatives to chicken. Foods.

[69] Gorman M., Moss R., Fisher C., Knowles S., Ritchie C., Schindell K., McSweeney M.B. (2023). Perceptions of plant-based fish among Atlantic Canadians. J. Food Sci..

[70] Aschemann-Witzel J., Peschel A.O. (2019). Consumer perception of plant-based proteins: The value of source transparency for alternative protein ingredients. Food Hydrocoll..

[71] Onwezen M.C., Bouwman E.P., Reinders M.J., Dagevos H. (2021). A systematic review on consumer acceptance of alternative proteins: Pulses, algae, insects, plant-based meat alternatives, and cultured meat. Appetite.

[72] Bayarri S., Martí M., Carbonell I., Costell E. (2012). Identifying drivers of liking for commercial spreadable cheeses with different fat content. J. Sens. Stud..

[73] Zhang X., Guo H., Zhao L., Sun W., Zeng S., Lu X., Cao X., Ren F. (2011). Sensory profile and Beijing youth preference of seven cheese varieties. Food Qual. Prefer..

[74] Ng M., Chaya C., Hort J. (2013). Beyond liking: Comparing the measurement of emotional response using EsSense Profile and consumer defined check-all-that-apply methodologies. Food Qual. Prefer..

[75] King S.C., Meiselman H.L. (2010). Development of a method to measure consumer emotions associated with foods. Food Qual. Prefer..

[76] Spinelli S., Masi C., Dinnella C., Zoboli G.P., Monteleone E. (2014). How does it make you feel? A new approach to measuring emotions in food product experience. Food Qual. Prefer..

[77] Song X., Perez-Cueto F.J., Bredie W.L. (2018). Sensory-driven development of protein-enriched rye bread and cream cheese for the nutritional demands of older adults. Nutrients.

[78] Giezenaar C., Orr R.E., Godfrey A.J.R., Maggs R., Foster M., Hort J. (2024). Profiling the novel plant-based meat alternative category: Consumer affective and sensory response in the context of perceived similarity to meat. Food Res. Int..

[79] Cardello A.V., Schutz H.G. (1996). Food appropriateness measures as an adjunct to consumer preference/acceptability evaluation. Food Qual. Prefer..

[80] Moss R., Barker S., Falkeisen A., Gorman M., Knowles S., McSweeney M.B. (2022). An investigation into consumer perception and attitudes towards plant-based alternatives to milk. Food Res. Int..

[81] Horgen K.B., Brownell K.D. (2002). Comparison of price change and health message interventions in promoting healthy food choices. Health Psychol..

[82] Paakki M., Kantola M., Junkkari T., Arjanne L., Luomala H., Hopia A. (2022). “Unhealthy= Tasty”: How Does It Affect Consumers’(Un) Healthy Food Expectations?. Foods.

[83] Scott A.G., Hunter S.C., Johnson B.J. (2022). Exploring the social norms regarding parents’ food provision in Australia using story completion methodology. Appetite.

[84] Templeton E.M., Stanton M.V., Zaki J. (2016). Social norms shift preferences for healthy and unhealthy foods. PLoS ONE.

[85] Reipurth M.F., Hørby L., Gregersen C.G., Bonke A., Cueto F.J.P. (2019). Barriers and facilitators towards adopting a more plant-based diet in a sample of Danish consumers. Food Qual. Prefer..

[86] Nicolás Saraco M., Blaxland J. (2020). Dairy-free imitation cheese: Is further development required?. Br. Food J..

[87] Grasso N., Alonso-Miravalles L., O’Mahony J.A. (2020). Composition, physicochemical and sensorial properties of commercial plant-based yogurts. Foods.

[88] Gupta M.K., Torrico D.D., Ong L., Gras S.L., Dunshea F.R., Cottrell J.J. (2022). Plant and dairy-based yogurts: A comparison of consumer sensory acceptability linked to textural analysis. Foods.

[89] Appiani M., Cattaneo C., Laureati M. (2023). Sensory properties and consumer acceptance of plant-based meat, dairy, fish and eggs analogs: A systematic review. Front. Sustain. Food Syst..

[90] Hoek A.C., Luning P.A., Weijzen P., Engels W., Kok F.J., De Graaf C. (2011). Replacement of meat by meat substitutes. A survey on person-and product-related factors in consumer acceptance. Appetite.

[91] Torrico D.D., Fuentes S., Viejo C.G., Ashman H., Dunshea F.R. (2019). Cross-cultural effects of food product familiarity on sensory acceptability and non-invasive physiological responses of consumers. Food Res. Int..

