# Supplementary material for: Consumer Expectations for Cream Cheese: A Category Appraisal Study in the United Kingdom with Dairy and Plant-Based Variants in Various Flavours
Source: Foods. 2025 Jan 29;14(3):445. doi: 10.3390/foods14030445 (PMC11816490; doi:10.3390/foods14030445)

SUPPLEMENTARY MATERIAL

PART SM1. Supporting table with the sample of 1093 participants and UK 2021 Census comparative data for selected participant characteristics.

Participant characteristics	Total Sample	UK Census*
**Gender**		
Male	47	49
Female	53	51
Other**	<1	<1
**Age bracket**		
18-29 years old	12	23
30-39 years old	20	21
40-49 years old	20	19
50-59 years old	18	21
60-69 years old	29	17
**Age group**		
18-45 years old	46	55
46-69 years old	54	45
**Location**		
South East	15	14
London	11	13
North West	11	11
East	9	9
Midlands	18	16
South West	9	9
Yorkshire and the Humber	9	8
North East	4	4
Scotland	8	8
Wales	4	5
Northern Ireland	2	3
**Ethnicity*** **		
British (English/Welsh/Scottish/Northern Irish)	87	
Black African	2	
Indian	3	
Other European	3	
Other	5	
Prefer not to answer	1	
**Dairy intake (incl., milk, yoghurt, and cheese)**		
I do not limit my dairy intake	73	
I limit my dairy intake but I still include dairy in my diet	27	

I do not eat dairy	0
**Reduction in dairy consumption at least 1 day a week**	
Yes	32
No	68

Notes.
*) This column reflects the available data from England and Wales (2021 Census) obtained from the Office for National Statistics (ONS), Northern Ireland (2021 Census) obtained from the Northern Ireland Statistics and Research Agency (NISRA), and Scotland (2022 Census) obtained from National Records of Scotland (NRS). **) Other gender refers to individuals whose gender identity is different from the sex registered at birth. ***) Total responses exceed 100% because multiple options can be selected.

PART SM2. A screenshot from the BWS task used in the online survey to elicit the expected liking for cream cheese and its PB alternatives, and the BIBD defining the combination of products in each BWS choice set.

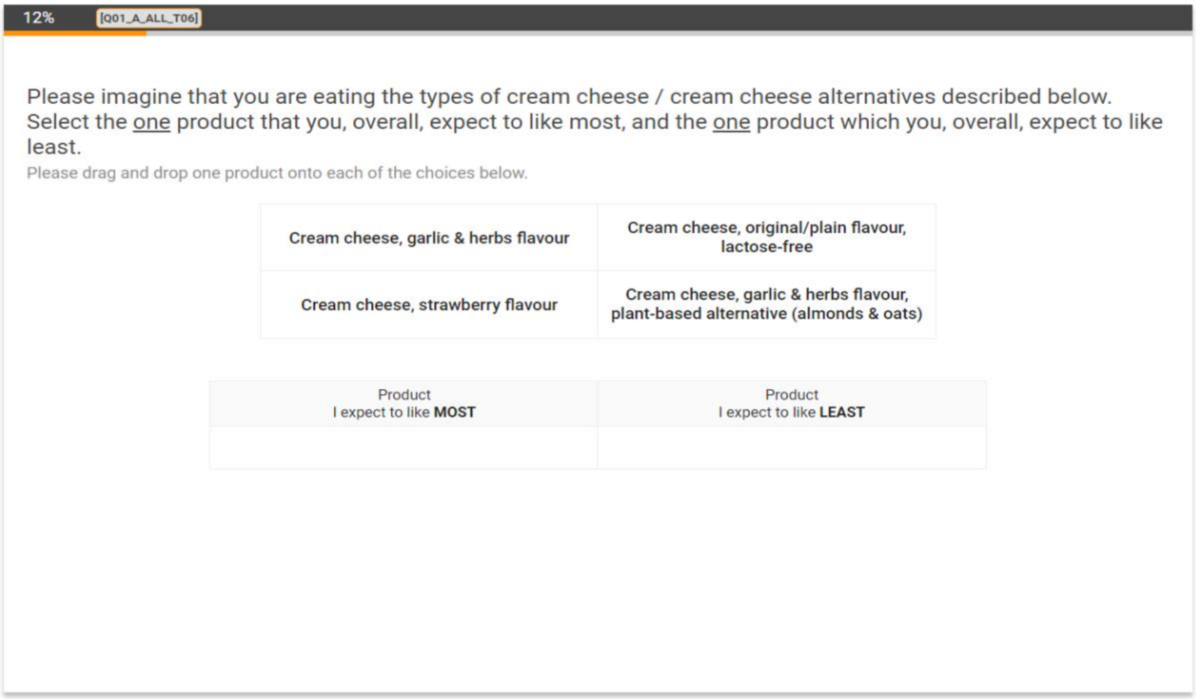

**BWS choice set**	**Option 1**	**Option 2**	**Option 3**	**Option 4**
Set 1	Object 1	Object 2	Object 4	Object 10
Set 2	Object 2	Object 3	Object 5	Object 11
Set 3	Object 3	Object 4	Object 6	Object 12
Set 4	Object 4	Object 5	Object 7	Object 13
Set 5	Object 5	Object 6	Object 8	Object 1
Set 6	Object 6	Object 7	Object 9	Object 2
Set 7	Object 7	Object 8	Object 10	Object 3
Set 8	Object 8	Object 9	Object 11	Object 4
Set 9	Object 9	Object 10	Object 12	Object 5
Set 10	Object 10	Object 11	Object 13	Object 6
Set 11	Object 11	Object 12	Object 1	Object 7
Set 12	Object 12	Object 13	Object 2	Object 8
Set 13	Object 13	Object 1	Object 3	Object 9

PART SM3. Number of participants per product stimuli in the 6-sample subset.

**Product descriptions**	**Short name in Figures/Tables**	**N in sample = 1222**	**N in sample = 1093**
Cream cheese, original/plain flavour*	Orig	203	186
Cream cheese, original/plain flavour, low-fat*	Orig_LowFat	204	183
Cream cheese, original/plain flavour, plant-based alternative (almonds & oats)*	Orig_PB_AO	202	180
Cream cheese, garlic & herbs flavour*	G&H	204	179
Cream cheese, garlic & herbs flavour, low-fat*	G&H_LowFat	204	186
Cream cheese, garlic & herbs flavour, plant-based alternative (almonds & oats)*	G&H_PB_AO	205	179

PART SM4. Data quality statement

The final sample of 1222 people was reached by approaching a total of 7082 people, of which 3256 did not satisfy the eligibility criteria, and 594 began but did not complete the survey. *Post hoc*, 25 people who had completed the survey were excluded because they had completed the survey very quickly (operationalised as faster than 1/3 of the median response time), which is linked to careless responding. A total of 88 people who completed the survey and passed the "speeder criterion" were also removed *post-hoc* because they provided the same score (i.e., flatlining) for all statements in a 10-item scale on food neophobia. A further 180 people were removed because their responses later in the survey indicated that they were intolerance to lactose (135) and did not eat dairy (45). Of the 2894 qualified participants, a group of 1717 people completed other tasks that were not related to the present research. The median time to complete the required tasks was 14.9 (IQR=10.2) min.

PART SM5. The ErrVarNorm (EVN) data quality index for BWS task (sustainable food choice motives)

According to Llobell et al. (2024), the ErrVarNorm index takes values between 0 and 1 and increases with the consistency of the respondent's answers.

$$\text{ErrVarNorm} = \frac{\sum_{i=1}^{p} x_i^2}{Max_{j=1}^{m}\{\sum_{i=1}^{p} x_i^2\}}$$

Where:

- M is the number of respondents
- p is the number of attributes in the BWS design
- x_i is the B-W score for attribute i.
- B-W scores (often referred to as BWS scores) are the 'best' frequency counts minus 'worst' frequency counts

PART SM6. Illustration of how inconsistent responding in a Case 1 BWS task influences the ErrVarNorm (EVN) index.

Consider a Case 1 BWS task with 11 objects (O01 to O11) implemented using a BIBD where each object appears 5 times. The table below shows responses from 5 participants. The light grey highlights for Persons B to E draw attention to differences in responses compared to Person A

Object	Person A	Person B	Person C	Person D	Person E
O01	5	3	4	3	5
O02	3	2	1	3	2
O03	2	2	1	2	2
O04	1	1	1	1	1
O05	0	1	1	0	1
O06	0	0	0	0	0
O07	0	-1	-1	0	1
O08	-1	-1	-1	-1	-1
O09	-2	-2	-1	0	-1
O10	-3	-2	-1	-3	-3
O11	-5	-3	-4	-5	-5
ErrVar	78	38	40	*58*	*72*
**EVN**	**1.00**	**0.49**	**0.51**	**0.74**	**0.92**

- Person A is fully consistent and always chooses the same object (here O01) as 'best' the 5 times it appears; he/she always also chooses the same object (here O11) as 'least' the 5 times it appears in the BWS task. Moreover, the consistency is also perfect for the following objects (second best, second worst, etc.) This results in EVN = 1.00.
- Person B has no strong preferences and does not consistently select one of the objects as 'best' and another as 'worst'. The most consistent response is for O01 which is selected as 'best' 3 of the 5 times it is presented. The other 2 times the object is presented, another object in the choice set is selected as 'best'. The type of responding reduces EVN to just below 0.5.
- Person C also displays inconsistent responding, but in a manner that is different to Person B. For Person C, O01 is selected as 'best' on 4 of 5 occasions, and O11 is selected as 'worst' on 4 of 5 occasions. Thus, this person displays clear preferences relative to these two objects, but an absence of preferences for the other 9 objects which are each selected once as either 'best' or 'worst'. This type of responding also reduces EVN, now to just above 0.5.
- Person D is more consistent that Persons B and C and has a response behaviour that is identical to Person A with two exceptions – O01 and O09. Rather than selecting O01 as 'best' every time it appears in the BWS task, Person D only selects O01 3 of 5 times. On the two occasions where O01 is not selected as 'best', O09 is selected instead. Because O01 has a major impact on Person D's preferences, the negative impact on EVN is relatively large and it reduces to 0.72.
- Person E has the EVN that is closest to 1.00 despite a response patterns that differs from Person A for 4 of 11 objects. The reason why the EVN reduction is small is that Person E's inconsistency pertains to objects that do not strongly drive preferences. The responses for O01 and O11 are identical to those from Person A.

PART SM7. The dendrogram following hierarchical cluster analysis on B-W scores for expected liking of the evaluated product stimuli (EF4). Comparison of solutions with 2, 3 and 4 clusters, lead to a final solution with 3 clusters (here shown as C1, C2, C3) based on the dissimilarity criterion. The horizontal line identifies which participants are allocated to which cluster.

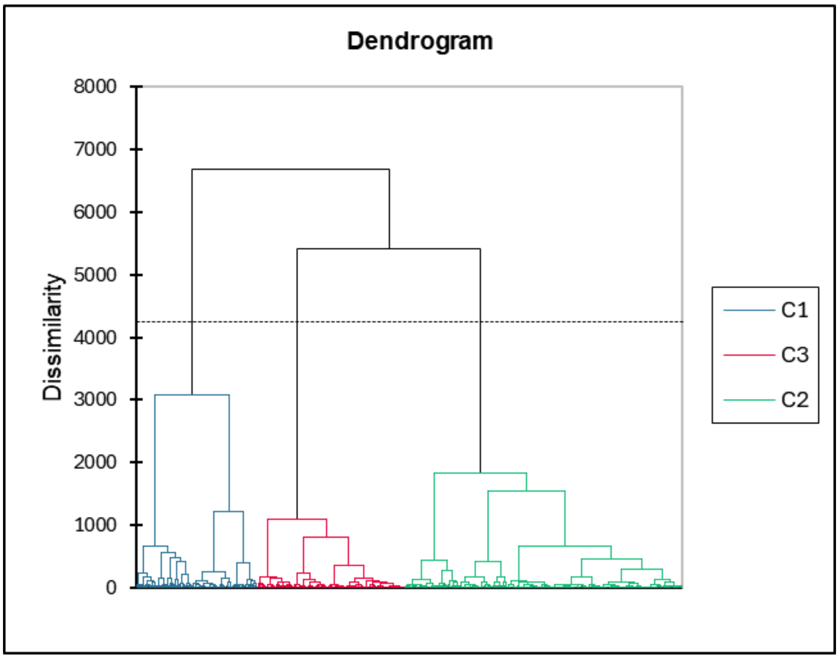

PART SM8. Results for EF1. Supplement to Figure 2a with labelling of all products and terms.

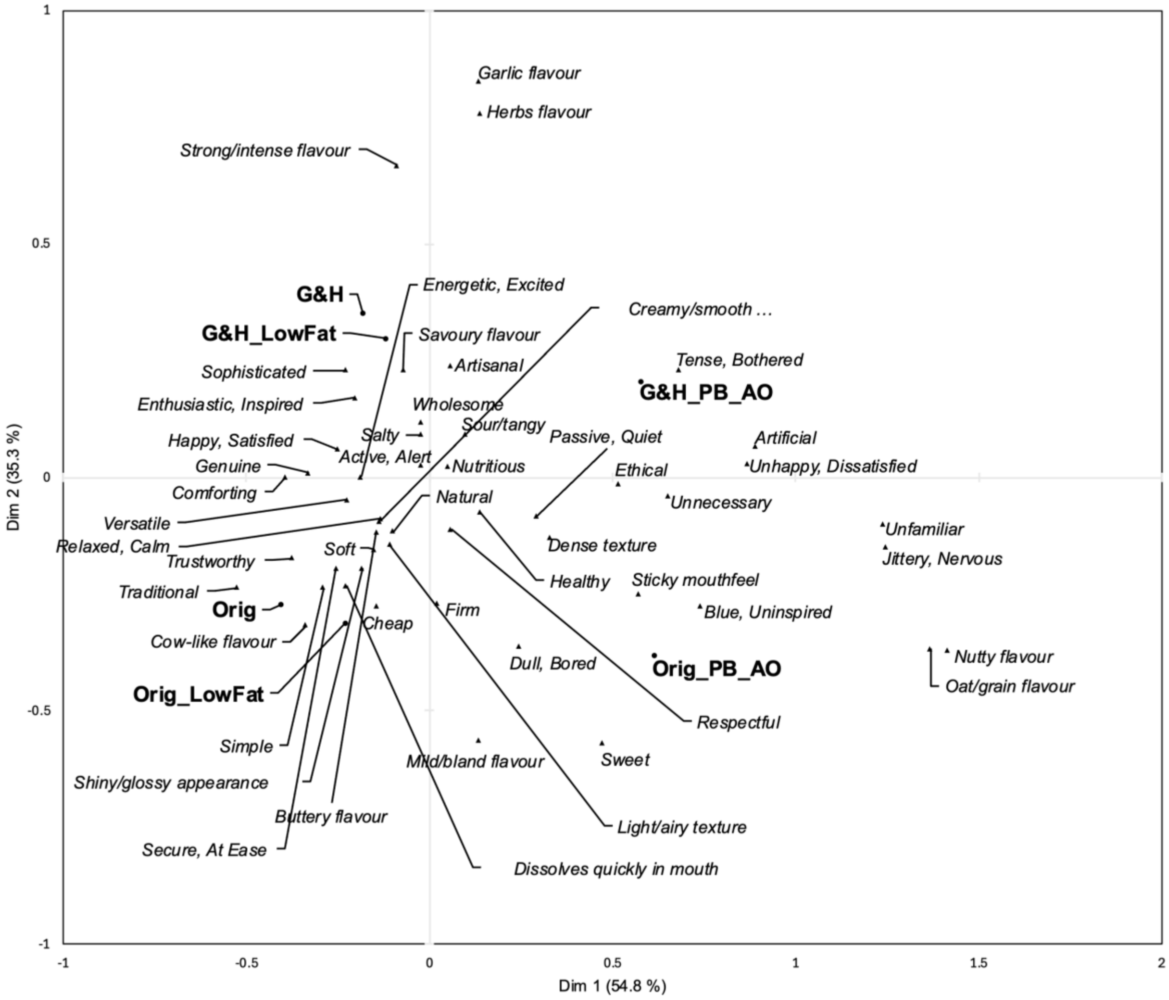

PART SM9. Results for EF1. Two-dimensional solutions following Correspondence Analysis based on expected sensory associations to the 6 stimuli in the product subset. A) biplot showing samples and terms. B) product space with 95% confidence ellipses around average variable positions.

A)

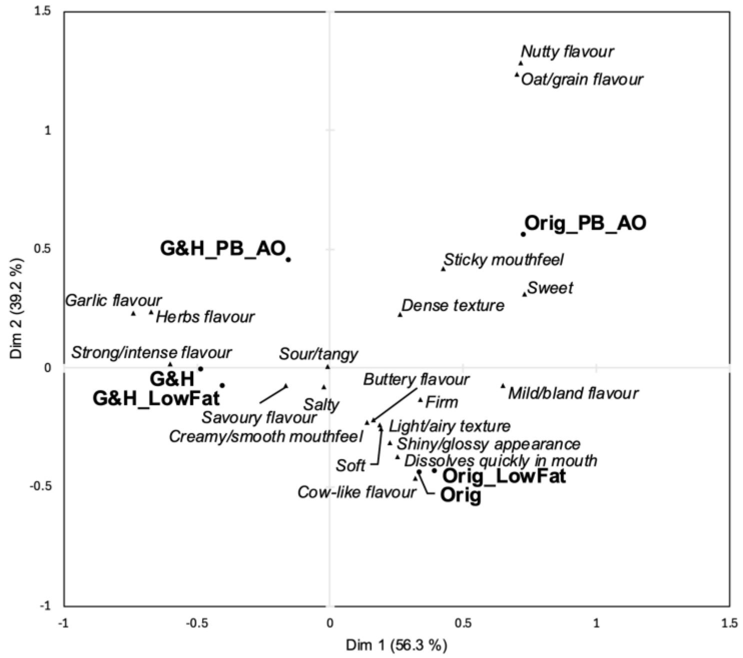

B)

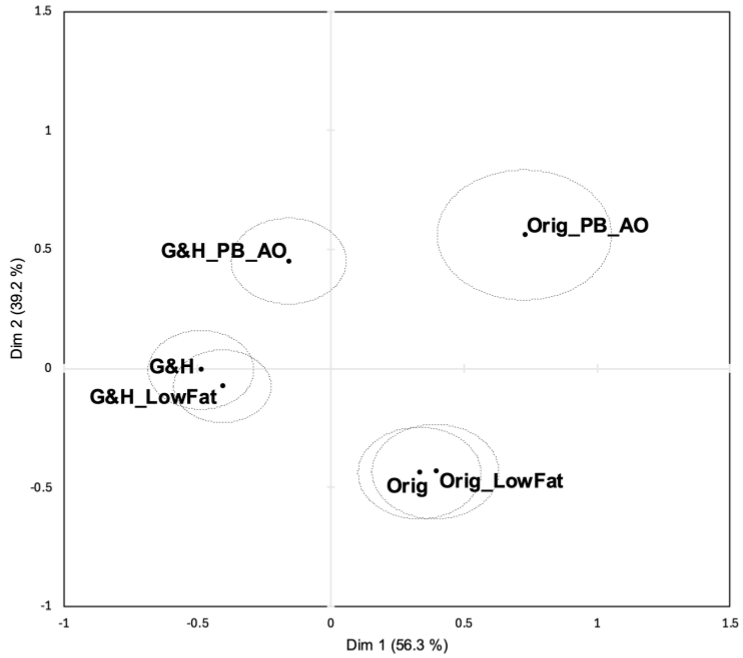

PART SM10. Results for EF1. Two-dimensional solutions following Correspondence Analysis based on expected emotional associations to the 6 stimuli in the product subset. A) biplot showing samples and terms. B) product space with 95% confidence ellipses around average variable positions.

A)

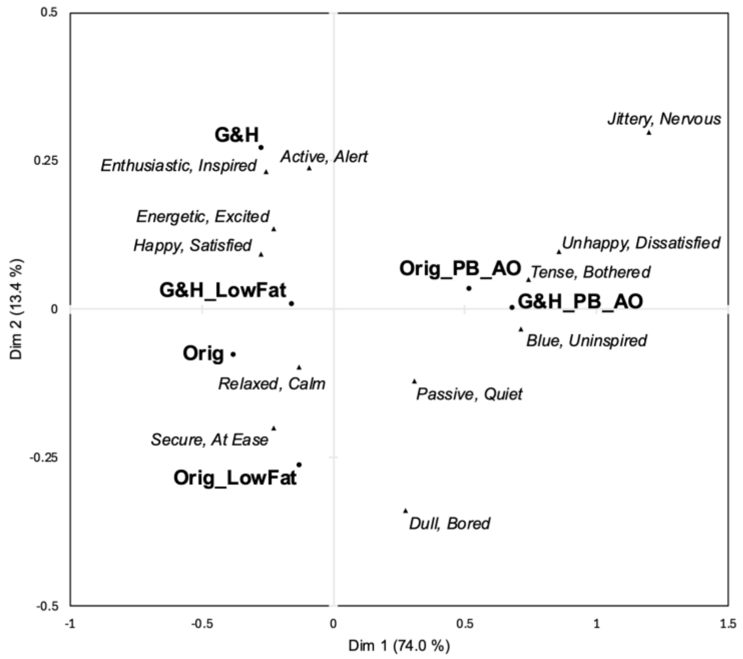

B)

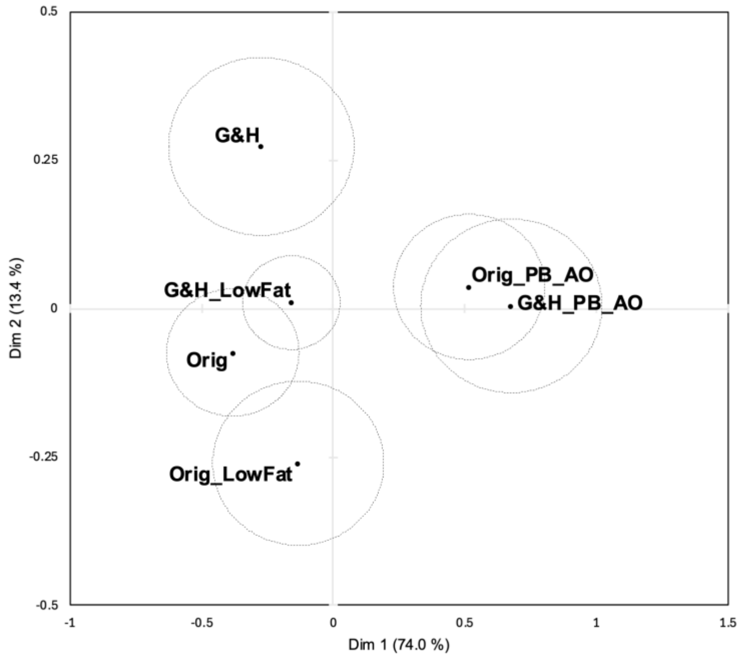

PART SM11. Results for EF1. Two-dimensional solutions following Correspondence Analysis based on expected conceptual associations to the 6 stimuli in the product subset. A) biplot showing samples and terms. B) product space with 95% confidence ellipses around average variable positions.

A)

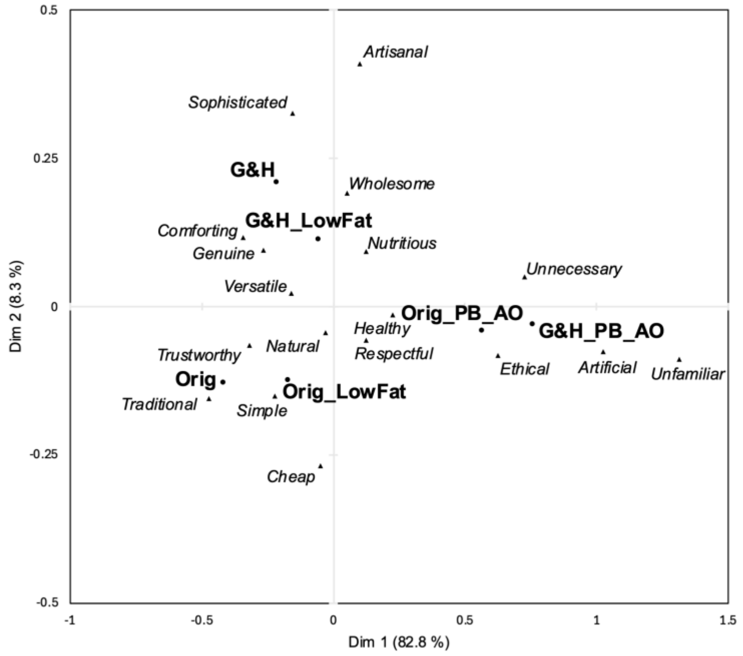

B)

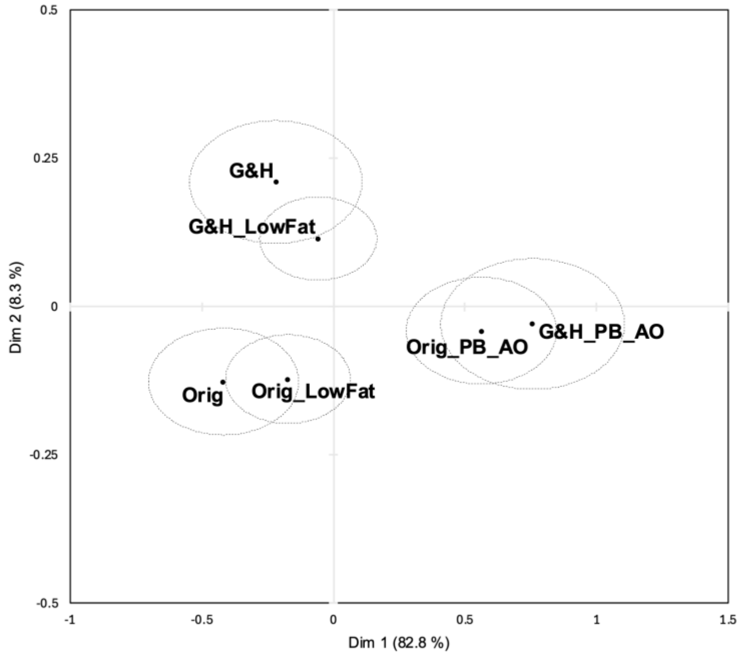

PART SM12. Results for EF4. A) Profile plot of mean expected product liking for the 13 product stimuli included in the research. Shown as B-W scores (+4 to -4) by cluster based on responses from 947 participants. B) Scatterplot of B-W scores for 12 product stimuli in Cluster 2 and Cluster 3 (salmon-flavoured sample excluded).

A)

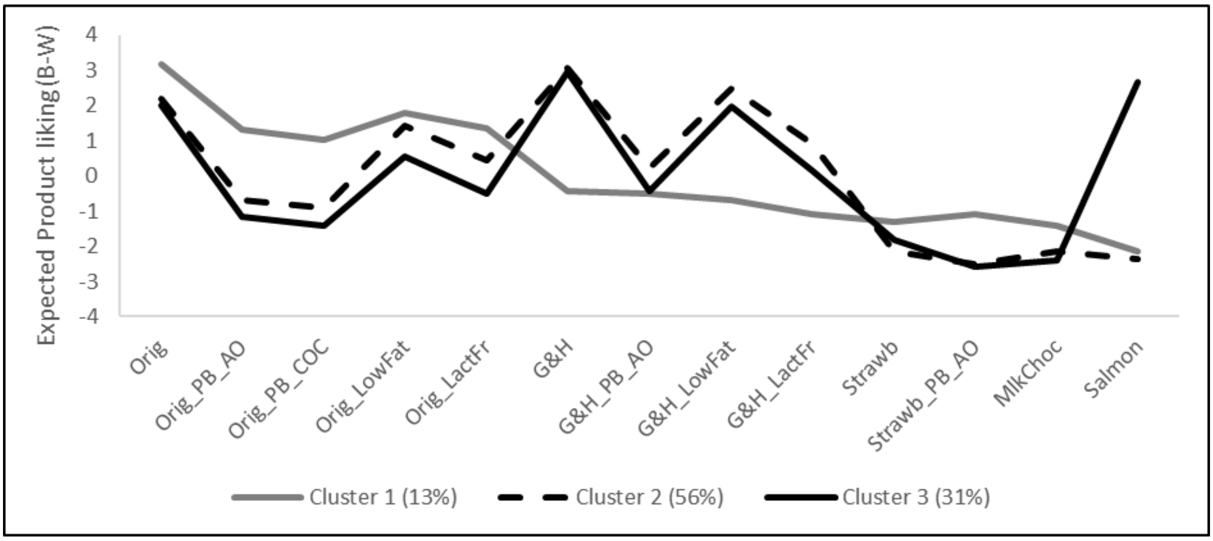

B)

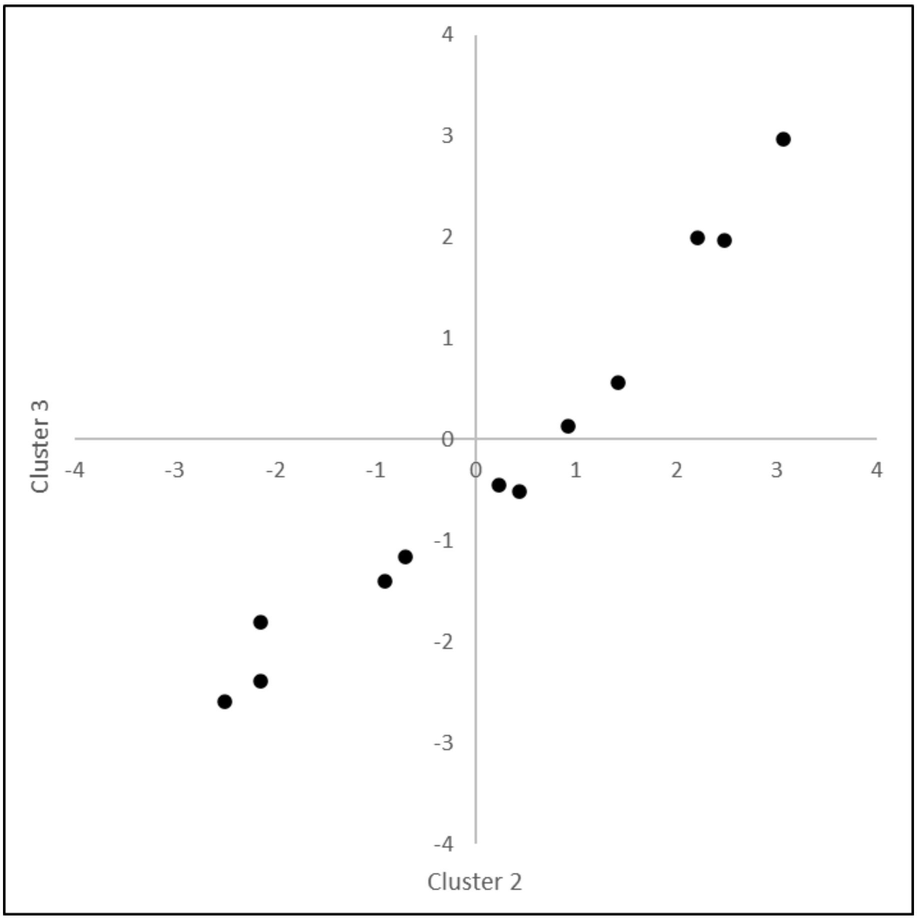

Supplement: Supplementary file 1 [file foods-14-00445-s001.zip › foods-3381408-supplementary.pdf]
